# Establishment and Characterization of an Epstein-Barr Virus–positive Cell Line from a Non-keratinizing Differentiated Primary Nasopharyngeal Carcinoma

**DOI:** 10.1158/2767-9764.CRC-23-0341

**Published:** 2024-03-04

**Authors:** Annie Wai Yeeng Chai, Shi Mun Yee, Hui Mei Lee, Norazlin Abdul Aziz, Pei San Yee, Marini Marzuki, Ka Wo Wong, Alan K.S. Chiang, Larry Ka-Yue Chow, Wei Dai, Teng Fei Liu, Lu Ping Tan, Alan Soo Beng Khoo, Kwok Wai Lo, Paul V.H. Lim, Pathmanathan Rajadurai, Howard Lightfoot, Syd Barthorpe, Mathew J. Garnett, Sok Ching Cheong

**Affiliations:** 1 Translational Cancer Biology Research Unit, Cancer Research Malaysia, Malaysia.; 2 Molecular Pathology Unit, Cancer Research Centre, Institute for Medical Research, Ministry of Health, Malaysia.; 3 Faculty of Medicine, Universiti Teknologi MARA, Malaysia.; 4 Department of Paediatrics and Adolescent Medicine, Li Ka Shing Faculty of Medicine, The University of Hong Kong, Hong Kong, P.R. China.; 5 Department of Clinical Oncology, Li Ka Shing Faculty of Medicine, The University of Hong Kong, Hong Kong, P.R. China.; 6 School of Biomedical Sciences, Li Ka Shing Faculty of Medicine, The University of Hong Kong, Hong Kong, P.R. China.; 7 Institute for Research, Development and Innovation and School of Postgraduate Studies, International Medical University, Bukit Jalil, Kuala Lumpur, Malaysia.; 8 Department of Medical Oncology, Sidney Kimmel Medical College, Thomas Jefferson University, Philadelphia, Pennsylvania.; 9 Department of Anatomical and Cellular Pathology, The Chinese University of Hong Kong, Hong Kong, P.R. China.; 10 Tung Shin Hospital, Malaysia.; 11 Subang Jaya Medical Centre, Malaysia.; 12 Jeffrey Cheah School of Medicine and Health Sciences, Monash University Malaysia, Malaysia.; 13 Wellcome Sanger Institute, Cambridge, United Kingdom.; 14 Department of Oral and Maxillofacial Clinical Sciences, Faculty of Dentistry, University of Malaya, Malaysia.

## Abstract

Nasopharyngeal carcinoma (NPC), a cancer that is etiologically associated with the Epstein-Barr virus (EBV), is endemic in Southern China and Southeast Asia. The scarcity of representative NPC cell lines owing to the frequent loss of EBV episomes following prolonged propagation and compromised authenticity of previous models underscores the critical need for new EBV-positive NPC models. Herein, we describe the establishment of a new EBV-positive NPC cell line, designated NPC268 from a primary non-keratinizing, differentiated NPC tissue. NPC268 can undergo productive lytic reactivation of EBV and is highly tumorigenic in immunodeficient mice. Whole-genome sequencing revealed close similarities with the tissue of origin, including large chromosomal rearrangements, while whole-genome bisulfite sequencing and RNA sequencing demonstrated a hypomethylated genome and enrichment in immune-related pathways, respectively. Drug screening of NPC268 together with six other NPC cell lines using 339 compounds, representing the largest high-throughput drug testing in NPC, revealed biomarkers associated with specific drug classes. NPC268 represents the first and only available EBV-positive non-keratinizing differentiated NPC model, and extensive genomic, methylomic, transcriptomic, and drug response data should facilitate research in EBV and NPC, where current models are limited.

**Significance:**

NPC268 is the first and only EBV-positive cell line derived from a primary non-keratinizing, differentiated nasopharyngeal carcinoma, an understudied but important subtype in Southeast Asian countries. This model adds to the limited number of authentic EBV-positive lines globally that will facilitate mechanistic studies and drug development for NPC.

## Introduction

Nasopharyngeal carcinoma (NPC) has striking geographical variations in incidence trends, and its age-standardized rate ranges from approximately 0.4 cases per 100,000 people per year in North America and Europe to >20 cases per 100,000 people per year in Southern China (1, 2). It is endemic to Southeast Asia and is the fifth most common cancer in Malaysia (3). Non-keratinizing NPC is the most prevalent subtype in endemic regions, comprising either differentiated or undifferentiated subtypes that are ubiquitously associated with Epstein-Barr virus (EBV; refs. 4, 5). In endemic regions, especially in China, the undifferentiated subtype accounts for up to 95% of all cases (6); however, in some countries, such as Malaysia and Thailand, this subtype may only account for 41%–77% of cases, while the non-keratinizing, differentiated subtype ranges from 17.5% to 41% (4, 5, 7, 8). Notably, in non-endemic regions, such as the United States of America, despite the rarity of the disease, the incidence of non-keratinizing differentiated NPC is increasing (9).

EBV infection in NPC cells is generally of latency type II, expressing a minimal subset of gene products, including EBNA1, LMP1/2, EBER1/2, BARF1, and BARTs miRNA (10). The involvement of EBV infection in dysregulating genetic, epigenetic, and cellular events underlying NPC pathogenesis has been well established (11–17). Exploiting EBV infection by either inhibiting its latent gene products or inducing lytic reactivation of EBV has been widely explored as therapeutic strategies (18–22). However, their clinical application is still limited, partly owing to an incomplete understanding of the cellular mechanisms underlying EBV pathogenesis and regulation of latent lytic switching (18). Furthermore, the scarcity of authentic EBV-positive NPC cell line models has been a major challenge in EBV and NPC research, hampering the identification and evaluation of novel therapies for the disease.

NPC cell lines are difficult to establish in culture (23). Moreover, many previously established NPC cell lines have lost their episomal EBV during prolonged culture and have major authentication issues (24). Until recently, C666-1, which was established from an NPC xenograft (25) was the only available EBV-positive undifferentiated NPC cell line. More recently, new EBV-positive undifferentiated NPC cell lines, including C17, developed from an NPC xenograft and NPC43 established from a nasopharyngectomized recurrent NPC, were established with the use of ROCK inhibitor (Y-27632; refs. 23, 26, 27). Despite this, the development of more cell lines, including those from the underrepresented subtypes and populations, is critical to appropriately model the genetic heterogeneity of the disease (23, 25).

Herein, we describe the successful establishment and characterization of the first EBV-positive NPC cell line derived directly from a primary non-keratinizing, differentiated NPC, designated NPC268. NPC268 is highly tumorigenic *in vivo* and produces infectious EBV particles upon lytic induction *in vitro*. This highly characterized model provides new insights into the disease and serves as a valuable resource for investigating the genomic, epigenomic, transcriptomic, and drug response landscape of NPC.

## Materials and Methods

### NPC Tissue Collection, Cell Line Establishment and Maintenance

The resected NPC tissue used for NPC268 cell line establishment was collected from a consented patient with Institutional Review Board approval. Tissue was collected in DMEM/F12 supplemented with 2% IU penicillin/streptomycin and 2% fungizone, and additional tissue samples were snap-frozen in liquid nitrogen. The tissue was washed, minced, and transferred into trypsin explant solution for disaggregation at 4°C overnight, and was further dissociated after incubation at 37°C the next day. The dissociated tissue was then subjected to conditional reprogramming, which involved the coculture of epithelial cells with irradiated 3T3-J2 feeder cells, in the presence of ROCK inhibitor (27). The NPC268 cell line was grown and established in FAD media supplemented with 5% FBS (Gibco), 100 IU Penicillin/Streptomycin (Pen/Strep; Gibco), 0.4 µg/mL hydrocortisone, and 4 µmol/L Y-27632. Details of cell line establishment and maintenance protocols are provided in the Supplementary Materials and Methods.

### Cell Culture

At passage 110 onward, for long-term culture, the NPC268 cell line was adapted to RPMI1640 media (Gibco) supplemented with 10% FBS, 100 IU Pen/Strep, and 4 µmol/L Y-27632. Growth rate and morphology were monitored alongside FAD-cultured cells of the same passage, and no visible differences in morphology were observed. C17, NPC38, NPC43, and NPC53 were maintained in RPMI1640 media supplemented with 10% FBS, 100 IU Pen/Strep, and 4 µmol/L Y-27632, while C666-1, HK1, EBV-negative Akata, and Namalwa cell lines were maintained in the same medium without Y-27632. All cell cultures were maintained at 37°C in an incubator with 5% CO_2_. All cell lines were authenticated by short tandem repeat profiling and regularly tested to confirm the absence of *Mycoplasma* contamination using the MycoAlert PLUS Mycoplasma Detection Kit (Lonza Bioscience).

### Karyotype and Cytogenetic Analysis

NPC268 cells in early and late passages were prepared for karyotyping as described previously (28). Briefly, cells were treated with 0.4 mmol/L of uridine and 5 µmol/L fluorodeoxyuridine for 15 hours at 37°C, released with 5-bromo-2-deoxyuridine (BrdU) for 7 hours at 37°C, and arrested at metaphases with colcemid treatment (10 µg/mL) for 22 minutes at 37°C. Fixed cells on the slides were stained using the trypsin-Giemsa technique, with at least 20 metaphases analyzed for each passage (28).

### Histologic Sample Preparation and IHC Staining

The protocols were adapted from Hoe and colleagues (2017; ref. 29). See the detailed description in the Supplementary Materials and Methods section. The antibodies and probes used, their respective detection systems, and antigen retrieval are listed in Supplementary Table S4.

### EBV Lytic Induction and B-cell Infection Using EBV from NPC268

Following transfection with the BZLF1 expression plasmid or treatment with suberoylanilide hydroxamic acid (SAHA; 10 µmol/L), sodium butyrate (NaBu; 6 mmol/L) or tiglian 12-O-tetradecanoylphorbol-13-acetate TPA (40 ng/mL), the expression of lytic markers Zta and Ea-D was determined by Western blotting. EBV-negative Akata cells were infected with EBV collected from conditioned media of NPC268 cells following transfection with the BZLF1 expression plasmid (see Supplementary Materials and Methods).

### Lysates Preparation and Western Blotting

Total cell lysates were processed and analyzed by Western blotting as described previously (ref. 30; see Supplementary Materials and Methods). The list of antibodies used is provided in Supplementary Table S5. Uncropped Western blot images can be found in Supplementary Fig. S7.

### Total RNA Extraction and qRT-PCR

Total RNA was extracted using TRIzol reagent (Thermo Fisher Scientific), according to the manufacturer's instructions. Real-time quantitative PCR was conducted as reported previously (ref. 30; see Supplementary Materials and Methods).

### EBV Detection by Copy-number Assay and FISH

Genomic DNA (gDNA) was extracted using the QIAamp DNA Mini Kit (Qiagen), according to the manufacturer's instructions. PCR amplification was conducted on a 7500 Real-Time PCR System with TaqMan Fast Advanced Master Mix (Applied Biosystems) using EBNA1 or β-globin primers and probes system, as reported previously (31). Standard curves for EBNA-1 and β-globin were run in tandem with the NPC268 DNA samples in duplicate. The gDNA from the Namalwa cell line was used for normalization. The EBV copy number (CN) per cell was reported as the ratio of the CN of the EBV genome to the number of cells equivalent to the input of NPC268 gDNA (1 cell = 2 copies of the β-globin gene). Information on the primers and probes used is provided in Supplementary Table S1.

FISH assay using a DNA probe that targets the Bam H1-W repeats of the EBV genome was conducted as described previously (32) on early- and late-passage NPC268 cells.

### *In Vivo* Tumorigenicity Study in Mice

Six female NOD-scid gamma (NSG) mice (NOD.Cg-Prkdcscid Il2rgtm1Wjl/ SzJ; The Jackson Laboratory) ages 11 weeks were inoculated with NPC268 cells (p39 and p129, *n* = 3 mice each) for xenograft establishment. Each mouse was injected subcutaneously with 10 million cells at a 1:1 ratio of cells in the media: Matrigel. Tumor volume was calculated as *width^2^ × length × 0.5* and the mice were sacrificed for tumor harvesting when the volume reached 1,000 mm^3^. All mice were housed, maintained, and used in accordance with institutional guidelines and protocols approved by the Animal Care and Use Committee, Ministry of Health, Malaysia [ACUC/KKM/02(16/2016)(19)].

### Whole-genome Sequencing

gDNA was extracted from cryosections of snap-frozen tumor tissue and late passage NPC268 (p118) cells using the Qiagen All Prep kit (Qiagen). Quality checks, library preparation, and sequencing services were provided by Novogene Co. Ltd. using Illumina HiSeq 2000 with 150 bp paired end sequencing (HiSeq-PE150). An average target coverage of 100× and 60× was achieved for the tumor tissue (NPC268_T) and cell line (NPC268_CL), respectively. The raw sequence reads were processed and aligned to the hg38 human reference genome, and basic bioinformatic analyses were provided by Novogene, including single-nucleotide variant (SNV)/insertion-deletion (INDEL)/structural variants (SV)/copy-number variants (CNV) calling, annotation, and statistics. Variant annotations were performed using the ANNOVAR tool, and alternative allele frequencies were reported on the basis of various databases, including the 1000 Human Genome, Exome Aggregation Consortium (ExAC), Genome Aggregation Database (gnomAD), and exome sequencing project (ESP).

The annotated SNV files of NPC268_T and NPC268_CL were subjected to stringent filtering to remove potential germline mutations and identify likely somatic SNVs. Only non-synonymous SNVs in the exonic regions and those with minor allele frequencies less than 0.0001 (0.01%) in the ExAC database were included. We further shortlisted the SNVs found in cancer driver genes (union of the IntOGen driver gene list and COSMIC Tier 1 cancer census gene dataset), as described in Sanger cancer dependency map (https://depmap.sanger.ac.uk/documentation/datasets/gene-annotation-mapping/#CancerDriverGenes).

### EBV Genome Analysis and Variant Calling

All reads from the NPC268_T and NPC268_CL whole-genome sequencing (WGS) datasets were mapped to the human reference genome GRCh38 using the Burrows-Wheeler Aligner (BWA, version 0.7.17, RRID:SCR_010910). Reads that mapped to “chrEBV” were extracted and remapped against the EBV type I reference genome (GenBank accession number: NC_007605.1).

Variant calling and filtering were performed following the best practices of the Genome Analysis Toolkit (GATK, version 3.8.1.0, RRID:SCR_001876). Additional filtering was applied to variants located in repetitive EBV regions. A total of 1,019 SNVs and 37 INDELs for NPC268_CL and 1025 SNVs and 38 INDEL for NPC268_T were retained for subsequent analyses. EBV variants were annotated using SnpEff (version 4.3t) according to the EBV type I reference genome.

A total of 211 publicly available FASTA files on published EBV genomes from NPC endemic regions (62 NPC and 142 healthy donors from Hong Kong; ref. 33), EBV-positive NPC cell lines, saliva, or biopsies (C666-1, NPC43, GD1, GD2, M81), and commonly reported non-NPC EBV strains, such as Akata and B95.8/Raji, were downloaded from GenBank (Supplementary Table S2). The FASTA files were mapped to the EBV type I reference genome using Minimap2 (version 2.12, RRID:SCR_018550) and variants were mapped using BCFtools (version 1.11, RRID:SCR_005227).

### EBV Genome–based Principal Component Analysis and Phylogenetic Tree

The 211 publicly available samples, NPC268_T and NPC268_CL, were consolidated for principal component analysis (PCA) and phylogenetic tree analysis. Sample filtering was carried out prior to PCA with the following criteria: (i) genotyping rate >90%, (ii) SNVs minor allele frequency >5%, and (iii) missingness per individual <20%. In total, 2,781 SNVs were retained after filtering. PCA was performed using PLINK software (version 1.90).

For phylogenetic tree analysis, multiple sequence alignment (MSA) of 213 sequences was performed using MAFFT (version 7). The resulting MSA FASTA file was trimmed using trimAl (version 1.2) to remove the poorly aligned regions. A maximum-likelihood tree was then constructed using a transversion model (TVM+F+R5) determined by the Model Finder utility in IQ-TREE (version 1.6.11).

### Whole-genome Bisulfite Sequencing

The gDNA of late passage cell lines was processed for bisulfite conversion and library preparation, and whole-genome bisulphite sequencing (WGBS) was performed and analyzed as described in ref. 34 and in Supplementary Materials and Methods. The methylation ratio of a CpG site/region is the number of cytosine reads divided by the sum of the cytosine and thymine reads. The human genome was segmented using a Methyl Kit (v.1.16.1) for global methylation pattern visualization (35).

### RNA Sequencing

Total RNA extracted was subjected to RNA sequencing (RNA-seq) using the Illumina HiSeq2000 platform and processed using the iRAP pipeline at the Wellcome Sanger Institute, as described previously (36).

### Gene Set Enrichment Analysis/Single-sample Gene Set Enrichment Analysis

Gene set enrichment analysis (GSEA) or single-sample GSEA (ssGSEA) was conducted using the GenePattern 2.0 tool (RRID:SCR_003201; Regents of the University of California, Broad Institute, Massachusetts Institute of Technology; ref. 37).

### Anti-Cancer Compound Screening

High-throughput cell viability screening was carried out on 339 compounds that were built from the anti-cancer drug library from the Genomics of Drug Sensitivity in Cancer, with additional curated compounds targeting the essential genes of head and neck cancer (30). A Fluid X-XRD-384 automated reagent dispenser (Sopachem Analytical, Belgium) was used to dispense the optimized cell number into a 1,536-well plate. After 24 hours postseeding, the Echo 555 acoustic liquid handling platform (Labcyte Inc.) was used for drug dispensing following a seven-point dose across a 1,000-fold dilution range. After 72 hours of drug treatment, cell viability was assessed using the CellTiter Glo 2.0 Assay (Promega), with luminescence reading measured using the SpectraMax Paradigm MultiMode plate reader (Molecular Devices).

### Statistical Analyses

All data are expressed as mean ± SD. Statistical significance analyses were performed using two-tailed Student *t* test with GraphPad PRISM software v9 (RRID:SCR_002798), unless stated otherwise.

### Data Availability Statement

Supplementary Dataset S1 – List of filtered SNVs list in NPC268 tumor and cell line
Supplementary Dataset S2 – RNA-seq–derived gene expression matrix
Supplementary Dataset S3 – JSON file containing drug response data
All above datasets are downloadable from figshare via https://doi.org/10.6084/m9.figshare.21747095.

The WGS, WGBS, and RNA-seq datasets generated during the current study are available from the corresponding author on reasonable request. The WGS data are deposited on European Genome-phenome Archive (EGA) under accession number: accession number EGAS00001007172. Request of access to raw data can be submitted to Cancer Research Malaysia (CRMY)-Translational Cancer Biology Research Unit DAC (sokching.cheong@cancerresearch.my). The WGBS data are available on Gene Expression Omnibus repository—GSE233426. Other data generated or analyzed during this study are included in this published article (and its Supplementary Data files).

### Code Availability

No unreported or custom code was used in this study. Codes used for data analysis are available upon request.

## Results

### Establishment of NPC268

**FIGURE 1:**
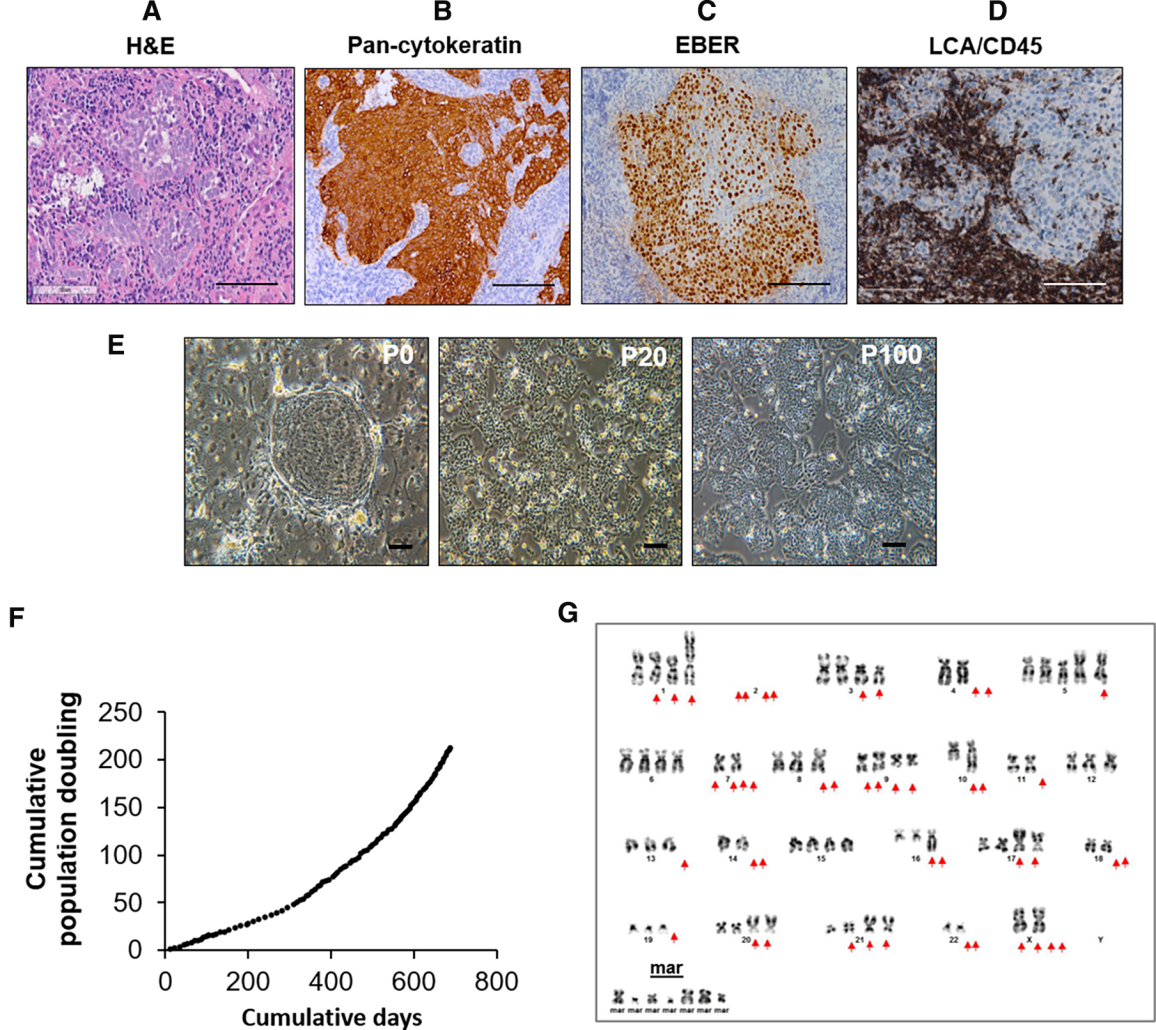
Establishment of a new EBV-positive NPC cell line, NPC268 from a non-keratinizing differentiated NPC primary tumor. **A,** H&E staining revealed NPC268 tumor as the non-keratinizing, differentiated NPC subtype. Scale bar: 100 µm. **B,** Epithelial origin was confirmed using pan-cytokeratin staining. Scale bar: 100 µm. **C,** EBER expression marked the EBV positivity in NPC268. Scale bar: 100 µm. **D,** LCA/CD45 staining reveals tumor-infiltrating lymphocytes in NPC268. Scale bar: 100 µm. **E,** Images of NPC268 cell line taken at passage 0, 20, and 100, respectively. Scale bar: 100 µm. **F,** Growth curve of NPC268 cell line, which has been cultured for >100 passages with no sign of senescence. **G,** Representative karyotype profile of the NPC268 cell line (at passage 108) revealed metaphases of hypotetraploidy with complex structural abnormalities.

The newly established NPC268 was derived from a tissue biopsy of the right nasopharynx of a treatment-naïve Malaysian Chinese female patient (39 years old) diagnosed in 2016 with primary, non-keratinizing, differentiated NPC (Fig. 1A). The presence of EBV in the epithelial tumor was confirmed by pan-cytokeratin and EBER staining (Fig. 1B and C). Tumor-infiltrating lymphocytes indicated by leukocyte common antigen (LCA)/CD45 staining were clearly seen in the tissue biopsy from which NPC268 was derived (Fig. 1D). The outgrowth of epithelial cells can be seen from the tissue within a week of explant (Fig. 1E, “P0”). The average doubling time of NPC268 was approximately 4.5 days and that of earlier passages was longer than that of late passages, suggesting that NPC268 comprised several different clones, and perhaps clones with a growth advantage may have outgrown others with a lower level of fitness. NPC268 cells were passaged over 200 population doublings without any sign of senescence (Fig. 1F). Cytogenetic analysis showed that the early passage NPC268 (p24) was dominated by hypodiploid clones (Supplementary Fig. S1A–S1D) with only one of the four clones being hypotetraploid, while at late passage (p108) the cells were all hypotetraploid (Fig. 1G; Supplementary Fig. S1E–S1G). Clones at late passage had many structural abnormalities, as described in Supplementary Fig. 1E–S1G. Such complex abnormalities, including loss of 17p, are generally considered to be indicators of poor prognosis, as observed in this patient who succumbed to the disease within 2 years of diagnosis. The epithelial origin of the NPC268 cell line was confirmed by IHC staining for pan-cytokeratin (Supplementary Fig. S1H). The authenticity of NPC268 was confirmed (Supplementary Table S3).

### EBV is Preserved in NPC268 and can be Induced into Lytic Reactivation

**FIGURE 2:**
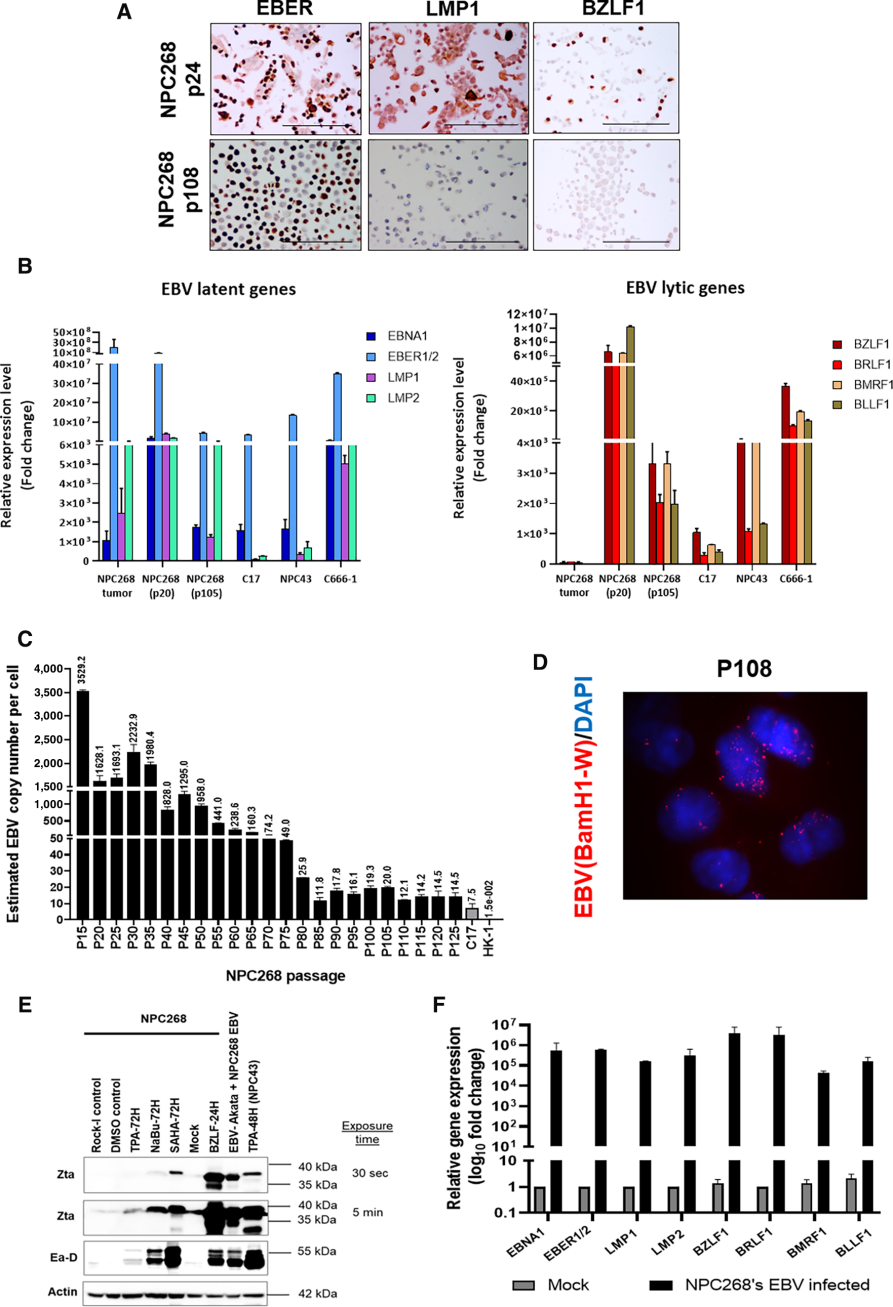
EBV is preserved in NPC268 and can be induced into lytic infection. **A,** EBER staining was positive in early and late passage of NPC268, but LMP1 and BZLF1 protein expressions were lost in the late passage. Scale bar: 100 µm. **B,** EBV latent and lytic gene expression profiles in NPC268 in comparison with other EBV-positive NPC cell lines. **C,** EBV CN in NPC268 has reduced following prolonged culture but stabilized at later passages, retaining about approximately 13 copies of EBV per cell. **D,** EBV-FISH assay confirming presence of EBV-positive cells in late passage of NPC268. **E,** EBV lytic genes-encoded protein expression in NPC268 induced by different methods. EBV-negative Akata cells infected with EBV derived from NPC268 expresses Zta and Ea-D, confirming infectious EBV production. NPC43 treated with TPA for 48 hours was used as positive control. **F,** qPCR analysis of the EBV-negative Akata cells showed substantial upregulation of various latent and lytic genes postinfection with the NPC268-derived EBV. Data are shown as mean ± SD (*n* = 2 biological repeats).

Despite being ubiquitously found in NPC, many NPC cell lines have lost their episomal EBV genome after long-term culturing. In NPC268, EBV is preserved even at late passage, likely in part due to the use of Y-27632 in suppressing lytic reactivation (Supplementary Fig. S2A) and cellular differentiation (23). EBER and LMP1 staining of early (p24) and late (p108) passage cells confirmed EBV positivity, although the proportion of LMP1-positive cells declined substantially at late passage (Fig. 2A). The *BZLF1* gene, indicative of lytic infection, can also be detected in a small subset of early passage cells but not in late passages. qPCR confirmed the high expression of EBV latent and lytic genes in early passages (Fig. 2B). Notably, although the lytic genes are being expressed in the tumor from which NPC268 is derived, their levels were generally low while that of the early passage NPC268 cell line showed high level of lytic genes expression. This observation confirms previous hypothesis that EBV lytic reactivation happens upon explanting the tumor to grow under *in vitro* condition. As this may hamper the establishment of cell line, the use of ROCK inhibitor to suppress its lytic reactivation is essential and hence the anticipated gradual decrease in EBV genes expression and CN is therefore observed. Nonetheless, EBV is preserved in NPC268 until late passages and their EBV latent/lytic gene expression levels were still comparable to those of other EBV-positive NPC cell lines.

The average EBV CN was higher at low passages, where the average CN was more than 1,000 copies per cell up to passage 35, but this gradually declined with increasing passages, as reported in other EBV-positive NPC lines (Fig. 2C). After passage 110, the EBV CN stabilized at an estimated 13 copies per cell, and the presence of EBV was confirmed using FISH (Fig. 2D).

The EBV latent-lytic switch is critical for the exploitation of lytic reactivation in cytolytic therapy for NPC (18). We showed that EBV can be readily induced in the lytic cycle, as shown by the upregulation of Zta (protein encoded by *BZLF1*) and Ea-D (protein encoded by *BMRF1*) in NPC268. SAHA was the most potent chemical agent in inducing EBV lytic reactivation (Fig. 2E; Supplementary Fig. S2B and S2C), whereas *BZLF1* overexpression was the most effective among all methods, where high levels of lytic proteins were observed as early as 24 hours posttransduction. Both western blot and qPCR analyses of the NPC268-derived EBV-infected Akata cells showed marked upregulation of Zta and Ea-D proteins, as well as various latent and lytic genes (Fig. 2E and F), providing evidence that EBV in NPC268 can be induced into productive lytic infection to produce highly infectious EBV particles.

### EBV Genome Analysis of NPC268 Reveals Resemblance with EBV Isolated from Southern Chinese NPC

**FIGURE 3:**
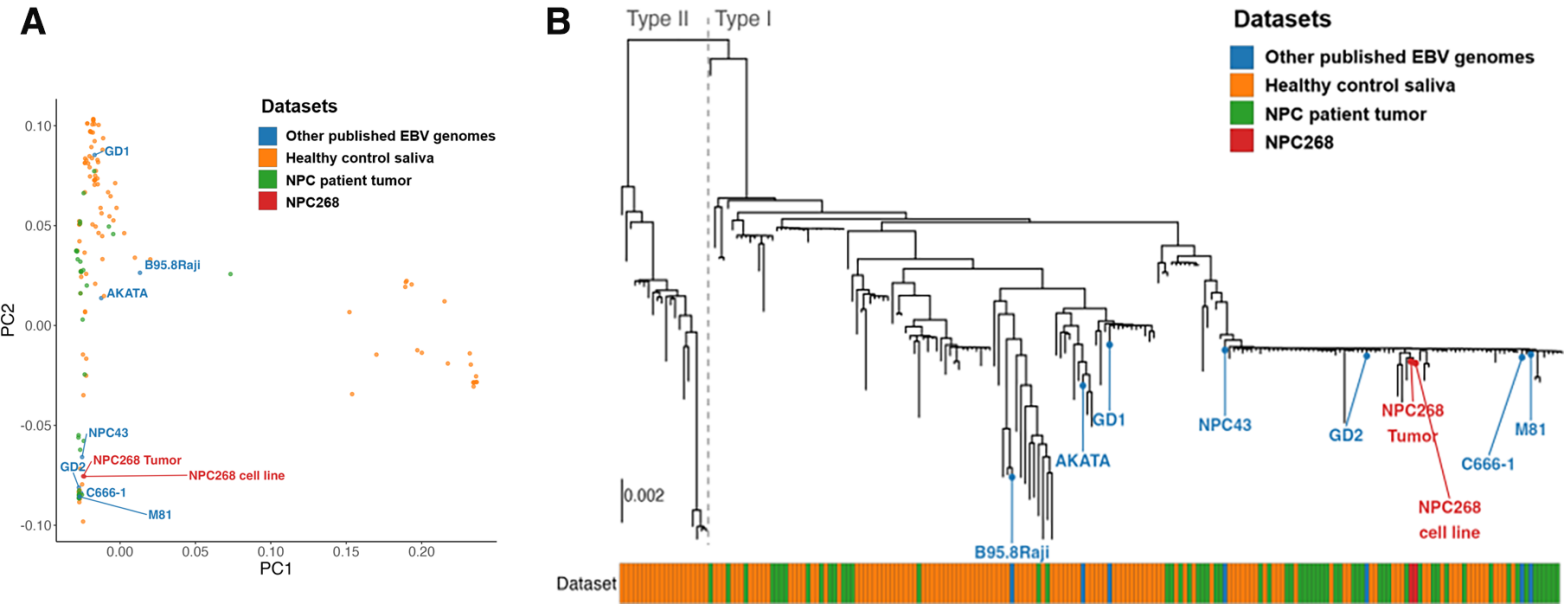
EBV genome analysis of NPC268 reveals resemblance with those commonly found in NPC from endemic regions. **A,** PCA of the 213 EBV genomes was performed. PC2 was plotted against PC1, each dot represents a previously published EBV genome [142 healthy control saliva (orange) and 62 NPC patient biopsies (green) from Hui et al. 2019; other published EBV genomes (blue) including those from NPC cell lines, saliva or biopsies (C666-1, NPC43, GD1, GD2, M81) and commonly reported non-NPC EBV strains such as Akata and B95.8/Raji. EBV from NPC268 tumor or cell line were indicated in red. **B,** Maximum-likelihood phylogenetic tree of 213 whole EBV genomes was performed. Type I and type II EBV are separated by the gray dotted line. Color panel at the bottom indicated the source of the EBV genomes.

The EBV genome from NPC268 was compared with 211 other published EBV genomes by PCA and maximum-likelihood phylogenetic tree analysis (Fig. 3A and B). We showed that the EBV from NPC268 is of type I EBV, and the EBV genomes of the NPC268 tumor and late passage cell line (red) clustered closely with the EBV strains reported by Hui and colleagues 2019 (green; ref. 33), and in close proximity to two other EBV-positive NPC cell lines, C666-1 and NPC43. Looking at a selected list of genes to further classify the EBV strain, we showed that NPC268 harbors the V-val subtype for EBNA1, and its LMP1 poses the 30 bp-deletion belonging to the China1 subtype, both of which are commonly associated with NPC in endemic regions (refs. 38–41; Table 1). Besides, in line with its strong lytic-reactivation capability, we found that the NPC268 EBV genome harbors the cancer-associated Zp-V3 subtype (42–44) within the *BZLF1* promoter, the 4 bp deletion downstream of *EBER2* variant (HKNPC-EBERvar; ref. 33) and lacks the *BALF2* variants that are associated with reduced lytic reactivation in epithelial cells (ref. 45; Table 1). In addition, NPC268 EBV also contains an *RPMS1* variant (G155391A) that results in a longer half-life of the RPMS1 protein (46).

**TABLE 1:** Summary of the NPC268 EBV classification and status of high-risk variants

EBV classification	NPC268
EBV type	Type I
EBNA1 subtype	V-Val
LMP1 30 bp-DEL	Present
LMP1 subtype	China1
Zp promoter type	Zp-V3
**High-risk variant from GWAS**	**NPC268**
*BALF2* (C162215A)	C (Ref)
*BALF2* (T162476C)	T (Ref)
*BALF2* (C163364T)	C (Ref)
*EBER4bp-DEL* (7187-7190delACTA)	HKNPC-EBERvar (Alt)
*RPMS1* (G155391A)	A (Alt)

NOTE: Summary of the subtype of EBV from NPC268 based on *EBNA1*, *LMP1* or Zp promoter and the list of high-risk variant status from GWAS studies, found in *BALF2*, *EBER2,* and *RPMS1*.

### NPC268 is Highly Tumorigenic and Demonstrates Lytic Activity *In Vivo*

**FIGURE 4:**
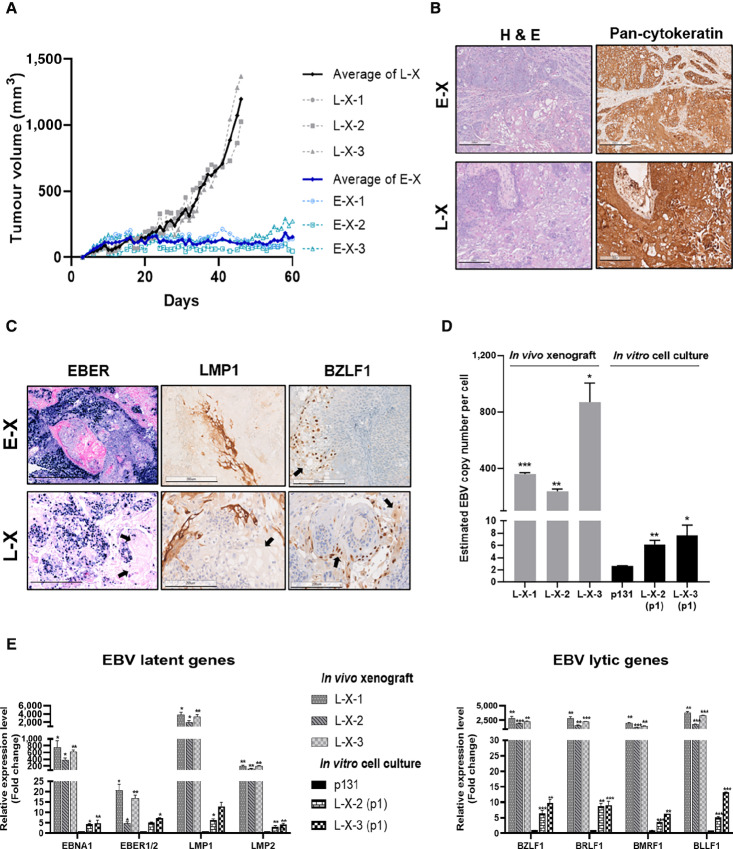
NPC268 is highly tumorigenic and demonstrate lytic activity *in vivo*. **A,***In vivo* growth curve of NPC268 mouse xenografts (early passage xenograft, E-X-1, E-X-2, E-X-3 and late passage xenograft, L-X-1, L-X-2, L-X-3). **B,** H&E and IHC staining of pan-cytokeratin of the mouse xenografts showing the non-keratinizing morphology of the xenografts, with some mixture of well-differentiated and poorly differentiated cells. **C,** Representative images of the EBER-ISH and IHC staining of LMP1 and BZLF1 on the xenografts. Positivity of these EBV markers confirmed the preservation of EBV in the late passage-derived xenografts. EBER and LMP1 staining were generally seen in the non-keratinizing, less differentiated cells while the BZLF1 were found in the more squamous-like, differentiated and keratinizing cells (black arrows), in which EBV are likely in permissive infection. **D,** Average EBV CNs per cell increased markedly in the xenograft tissue *in vivo*, compared with the *in vitro* cultured cells before (p131) and after [L-X-2 (p1), L-X-3 (p1)] xenograft transplantation. **E,** qPCR analysis of the EBV latent and lytic genes showing significant upregulation in the xenografts compared with those from the *in vitro* cultured cells. Data are shown as mean ± SD (*n* = 2 biological repeats). Two-tailed, unpaired Student *t* tests were performed for L-X xenografts, comparing with p131 *in vitro* cell culture; for L-X-2 (p1)/L-X-3 (p1) *in vitro* cell culture, comparisons were made with corresponding xenografts. *, *P* < 0.05; **, *P* < 0.005; ***, *P* < 0.001.

The ability of NPC268 to grow as spheroids and xenografts *in vivo* can extend its utility and applications. Late passage NPC268 formed spheroids of more than 100 µm in size after 28 days in soft agar culture (Supplementary Fig. S3A). We subcutaneously injected 10 million NPC268 early (p39; E-X) and late passage cells (p129; L-X) into the flank of 3 NSG mice each. Palpable tumors could be seen within a week and reached 1,000 mm^3^ in an average of 45 days for late passage NPC268 (Fig. 4A; Supplementary Fig. S3B); On the other hand, early passage NPC268 grew much slower (Fig. 4A) and remained on average below 200 mm^3^ at 60 days.

Hematoxylin and eosin (H&E) staining indicated that the xenografts consisted of both non-keratinizing, less differentiated cells and more keratinizing, squamous-like differentiated cells (Fig. 4B). Mixed histologic types of NPC were common in clinical specimens (47). Pan-cytokeratin and EBER stains confirmed the epithelial origin and EBV positivity, respectively (Fig. 4B and C). Consistent with the *in vitro* findings, EBER and LMP1 staining were much more intense among early passage xenografts. Notably, we found that the proportion of more keratinizing, squamous-like differentiated cells were much higher among L-Xs (average of 75%), compared with about 20% in E-Xs. As previously reported in clinical samples, we observed that the intensity of EBER staining decreases in areas of differentiation (47). LMP1 staining found in the periphery of undifferentiated cells was membranous and of various intensities. Numerous differentiation pearls of squamous cells (marked with black arrows) were also observed across the xenograft tissues, being more prevalent in L-Xs (Fig. 4C). As the cells mature, EBV converts from latency to permissive infection, as supported by the staining of the lytic marker BZLF1, which is more intense among the cells of the superficial layer where keratinization occurs (Fig. 4C). Evaluation of EBV CN under *in vitro* and *in vivo* conditions showed that the *in vivo* xenografts exhibited significant upregulation of EBV CN, while the *in vitro* cultured cells before and after inoculation showed much lower EBV CN (Fig. 4D). qPCR showed that both latent EBV and lytic genes were significantly upregulated in xenograft tissues (Fig. 4E).

### WGS Revealed NPC268 as a Genomically Unstable Tumor

**FIGURE 5:**
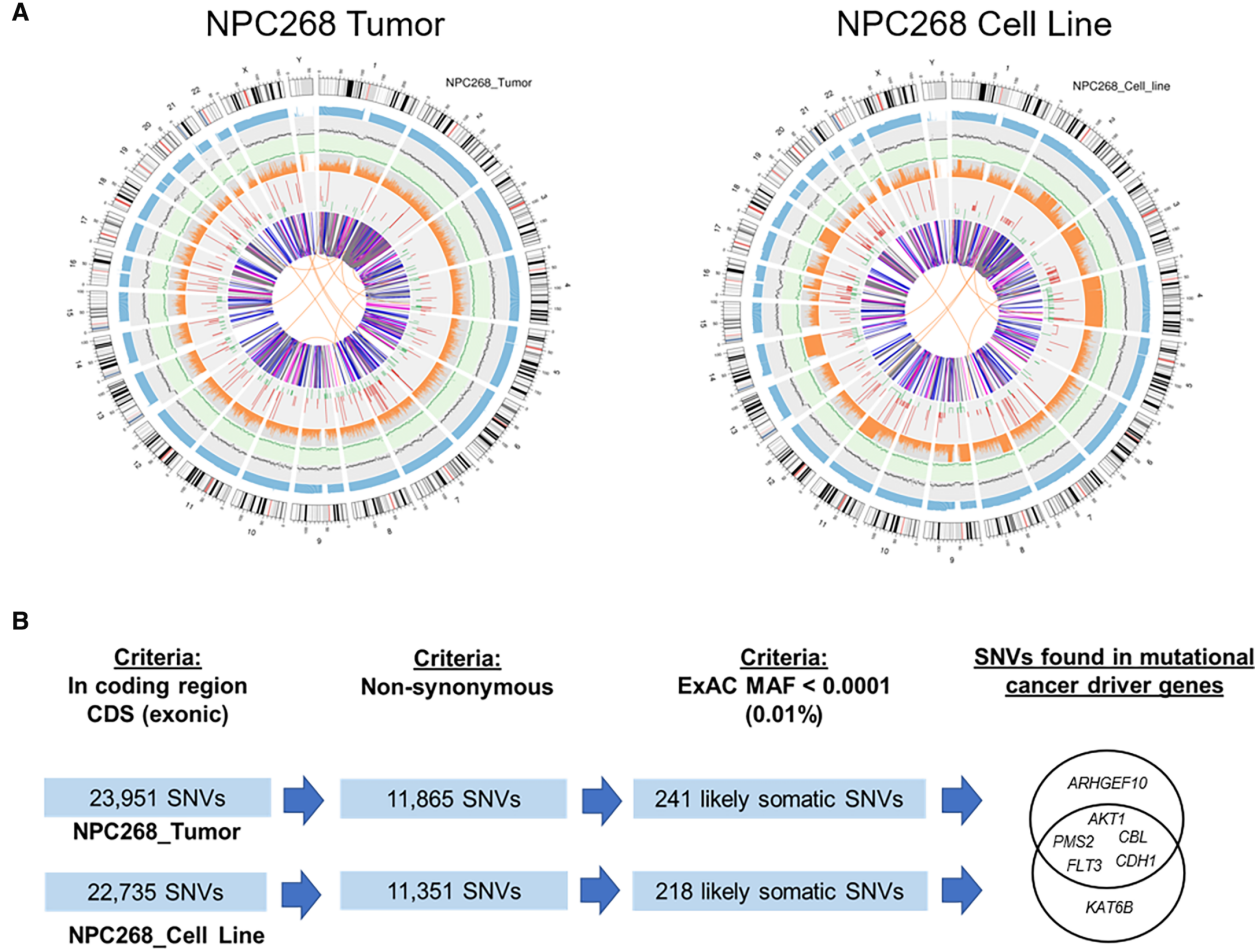
NPC268 is characterized by a high number of SVs and showed enrichment in immune-related gene expression. **A,** Circos plot showing an overview of genomic aberrations in NPC268 as revealed by WGS analysis. From outer to inner rings: (1) chrome information; (2) read coverage histogram per 0.5 Mbp region; (3) INDEL density scatter plot per 1 Mbp region; (4) SNV density scatter plot per 1 Mbp region; (5) proportion of homozygous SNV (ORANGE) and heterozygous SNV (GRAY) in histogram per 1 Mbp region; (6) CNV inference—gain (RED), loss (GREEN); (7) SVs—translocation (ORANGE), insertion (GREEN), deletion (GRAY), duplication (PINK), inversion (BLUE). **B,** Shortlisting strategy to identify potential pathogenic driver mutations in NPC268, focusing on those SNVs found in mutational cancer driver genes.

An overview of the genomic landscapes of NPC268 (late passage cell line and tumor) is depicted by Circos plots, where the cell line was shown to be highly recapitulative of the tumor (Fig. 5A). Notably, greater LOH and CNVs were observed in the cell line than in the tumor.

Comparing with C666-1 (48), NPC268 has a higher number of SNVs [∼3.8 million (M) in the tumor, ∼3.6M in the cell line vs. C666.1 at ∼2.3M]. Considering all the non-synonymous SNVs in the coding region of the tumor tissue and cell line, NPC268 had a high tumor mutational burden (TMB), estimated at approximately 395 mut/Mb (NPC268_T) and 378 mut/Mb (NPC268_CL). INDELs were observed at a slightly lower frequency (∼0.9M in NPC268_T, ∼0.8M in NPC268_CL) in NPC268 compared with C666-1 (∼1M); whereas NPC268 has a higher number of SVs (∼12k in NPC268_T; ∼9.3k in NPC268_CL) compared with C666-1 (∼5.8k; Supplementary Table S6). Consistent with karyotype observations, NPC268 harbors complex chromosomal rearrangements and many SVs, indicating that it is highly genomically unstable. Furthermore, gamma H2A.X can be readily detected in NPC268 cells (Supplementary Fig. S4A). Notably, there was an apparent loss of chromosome 2 in the karyotype analysis, which was reflected by many deletions, inversions, and duplications within chromosome 2 from the genomic analysis, as reflected in the Circos plot. Of interest, several mismatch repair genes (MMR), including *MSH2*, *MSH6,* and *PMS1* on chromosome 2, all fall within the chromosomal arm with SVs indicative of the presence of deletions, inversions, or duplications. Sequencing reads in this region show chaotic and complex rearrangements, suggesting a possible chromothripsis event involving chromosome 2, characterized by a large number of breakpoints (49, 50).

After stringent filtering criteria and focusing on annotated cancer driver genes, seven SNVs were identified in *AKT1* (p.E17K), *PMS2* (p.L47V), *CBL* (p.D772E), *FLT3* (p.D7G), *CDH1* (p.R124H), *ARHGEF10* (p.I175V), *KAT6B* (p.A854T; Fig. 5B; Supplementary Table S7; Supplementary Dataset S1). The driver mutation at E17K in *AKT1* is a biomarker of response toward targeted drugs in PI3K/AKT-mutated solid tumors (51, 52). In addition, the SNV in the MMR gene *PMS2* (p.L47V) falls within its ATPase region and might result in its loss of function. Notably, *PMS2* mutation was also found in C666-1 (53) while mutation of *KAT6B* has also been reported in NPC, albeit at a different site (54).

We also found that NPC268 consistently harbors CN losses in three chromosomal arms (Supplementary Table S8) in both tumor and cell line, including the 9p21.3 co-deletion of *CDKN2A* and *MTAP* which is a common event in NPC, implying that NPC268 could be vulnerable to MAT2A inhibitors (55). Other key CN losses include the 4q13.2 and 14q22.2 region affecting multiple genes, while there are focus CN gains in *CCDC186* (10q25.3), *TMEM216* (11q12.2), and *GCSH* (16q23.2), found with more than 10 CN in both NPC268_T and NPC268_CL. However, these CN alterations have not been previously reported in NPC.

### NPC268 Resembles the HypoNPC Subtype and Showed Enrichment in Immune-related Gene Expression

**FIGURE 6:**
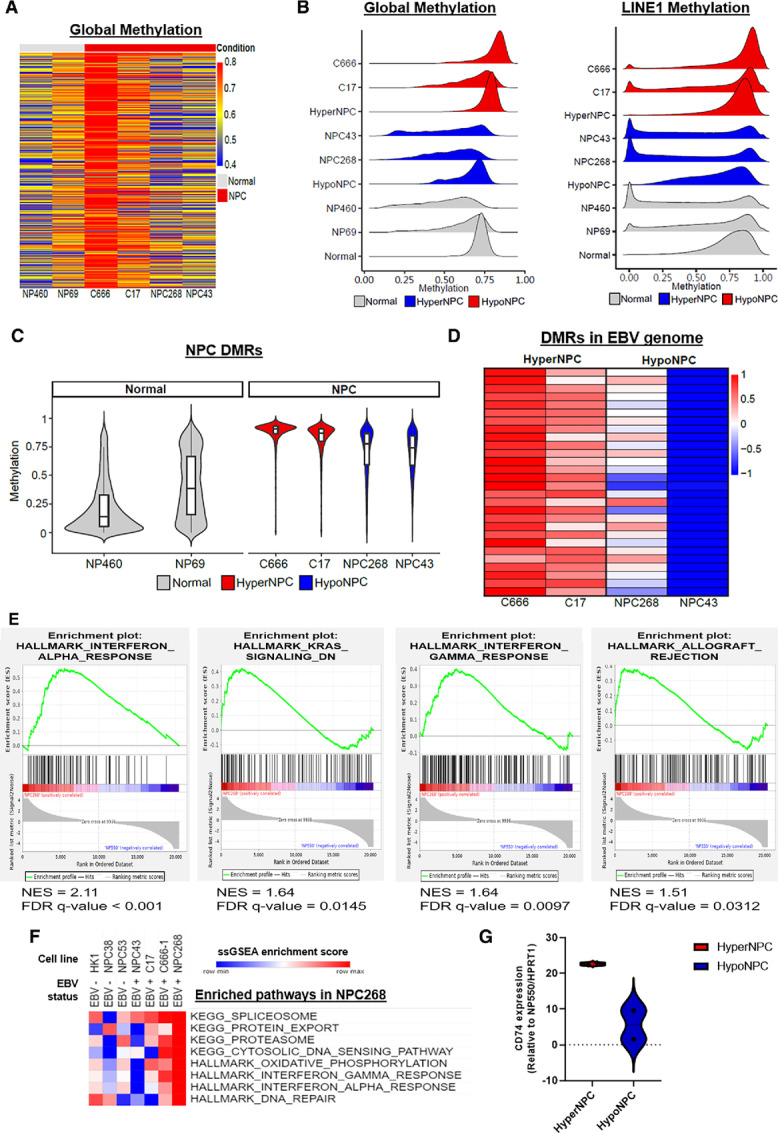
NPC268 resembles the HypoNPC subtype and showed enrichment in immune-related gene expression. **A,** WGBS analysis revealed the methylation level of NPC268, resembling that of HypoNPC subtype. Heat map shows the global methylation levels of the human genome in nasopharyngeal epithelial cell lines and EBV-positive NPC cell lines. The human genome was equally segmented into consecutive tiles of 1 Mbps, and a methylation average is calculated for each window. The heat map color scale denotes the absolute methylation level. **B,** Ridge plot shows the global methylation levels of the human genome in the 1 Mbps windows (left plot) and the LINE1 methylation level (right plot). Normal, HypoNPC, and HypoNPC were the adjacent non-cancerous nasopharyngeal tissue, HypoNPC tumor, and HyperNPC tumor derived from patients with NPC, respectively (Chow et al. 2022). Like NPC43, NPC268 has regions with low methylation levels compared with the normal clinical specimen, normal immortalized cell lines and HyperNPC. The unmethylated peak (at “0”) of LINE1 surrogate marker further support the global hypomethylation status. **C,** Violin plots showing the methylation levels in specific differentially methylated regions (NPC-DMR) identified in Chow et al. 2022. These NPC DMRs were hypermethylated in the NPC clinical tissues compared with the non-cancerous adjacent tissues. **D,** Heat map shows the methylation average of the DMRs between HyperNPC and HypoNPC clinical specimens in the EBV genome. NPC268 and NPC43 showed lower methylation levels, similar to HypoNPC whose methylation levels in these regions were lower compared with the HyperNPC. The heat map color scale denotes the Z-score of the methylation levels. **E,** GSEA of NPC268 against NP550 revealed upregulation of immune-related hallmark pathways. **F,** Heat map snapshot of KEGG or Hallmark pathways where NPC268 was found to have the highest ssGSEA enrichment scores. **G,** RNA-seq expression of CD74 is markedly higher in HyperNPC cell lines (NPC268, NPC43) compared with HypoNPC cell lines (C666-1, C17).

Epigenetic dysregulation is a common event that contributes to NPC pathogenesis. Two epigenome subtypes of NPC were recently described on the basis of clinical specimens: HyperNPC and HypoNPC, which are globally hypermethylated and globally hypomethylated, respectively in comparison with normal nasopharyngeal tissues (34). Compared with normal nasopharyngeal tissue and cell line (NP69/NP460), NPC268 showed low global methylation levels, resembling that of NPC43, a representative line of the HypoNPC subtype (ref. 34; Fig. 6A and B). Lower methylation levels of long-interspersed element-1 (LINE1), a surrogate marker of global DNA methylation, confirmed its global hypomethylation status (Fig. 6B). Also corroborating with the evidence of DNA damage and genomic instability in NPC268, we found that it expressed high levels of LINE1 RNA (Supplementary Fig. S4B), which is reflective of high LINE1 retrotransposon activity (56). The methylation levels at NPC-specific differentially methylated regions (NPC-DMR; DMRs in the human genome between NPC tumors and non-cancerous adjacent tissue; ref. 34) were higher in all four EBV-positive NPC cell lines than in the two nasopharyngeal epithelial cell lines (Fig. 6C). In the DMRs between HyperNPC and HypoNPC in the EBV genome that were previously identified (34), NPC268 and NPC43 showed lower methylation levels than C666-1 and C17 (Fig. 6D). These results collectively suggested that NPC268 (and NPC43) are representative lines of the HypoNPC subtype.

RNA-seq was performed on the late passage NPC268 cell line and compared with the nasopharyngeal epithelial cell line NP550 (Supplementary Dataset S2). GSEA showed significant upregulation of several immune-related HALLMARK pathways, including the “Interferon alpha response” [normalized enrichment score (NES) = 2.11; FDR *q*-value < 0.001]; “Interferon gamma response” (NES = 1.64; FDR *q*-value = 0.0097), “KRAS signaling DN” (NES = 1.64; FDR *q*-value = 0.0145); “Allograft rejection” (NES = 1.51; FDR *q*-value = 0.0312; Fig. 6E).

When comparing NPC268 with other NPC cell lines, we also found that NPC268 had the highest enrichment scores for several immune-related hallmark pathways, as shown by the ssGSEA score heat map (Fig. 6F; Supplementary Fig. S5A). The detection of micronuclei presents in NPC268 cells further confirm the activation of the IFN signaling in NPC268 (Supplementary Fig. S5B). The MHC class I molecules, including HLA-A and HLA-B/C, are expressed in NPC268, suggesting functional antigen presentation machinery (Supplementary Fig. S5C). HyperNPC was described to show upregulates *CD74*, a marker associated with immune exhaustion. We found that NPC268 and NPC43, two representative HypoNPC lines, had relatively lower expression of *CD74* (Fig. 6G).

A table summarizing the key differences and unique features of NPC268 when compared with the other three EBV-positive NPC lines was included as Supplementary Table S9.

### Drug Sensitivity Profile of NPC268 and Other NPC Cell Lines Show Preferential Sensitivity of NPC268 Toward BCL2 Inhibitors

**FIGURE 7:**
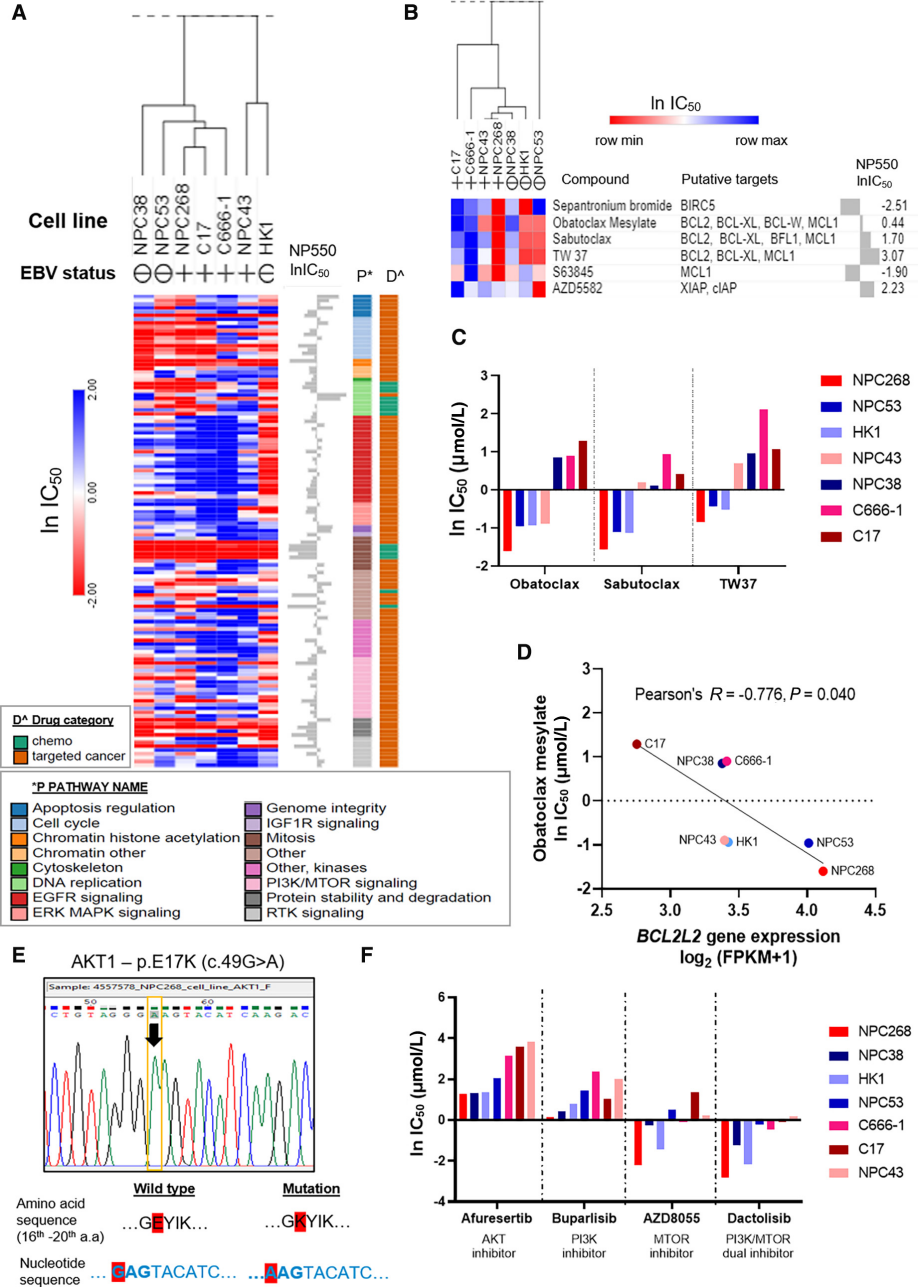
Drug sensitivity profile of NPC268 and other NPC cell lines show preferential sensitivity of NPC268 toward BCL2 inhibitors. **A,** High-throughput screening on 339 drugs was performed on NPC268 and other NPC cell lines (EBV-negative: NPC38, NPC53, HK1; EBV-positive: NPC43, C666-1, C17). Heat map shows the drug sensitivity of the seven NPC cell lines for the 125 active drugs (at least one cell line with IC_50_ <1 µmol/L). **B,** Heat map shows the drug sensitivity toward the six active drugs targeting apoptosis pathways. **C,** NPC268 is the most sensitive NPC line toward drugs targeting the antiapoptotic proteins such as obatoclax, sabutoclax, and TW37. **D,***BCL2L2* gene expression is found to be significantly correlated with sensitivity toward obatoclax mesylate, an inhibitor of *BCL2L2* (Pearson *R* = −0.776, *P* = 0.040). **E,** Sanger sequencing confirmed the E17K mutation in *AKT1* gene, showing an “A” instead of reference allele “G”, leading to a change in amino acid of glutamine (E) to lysine (K) at the 17th amino acid position. **F,** Bar charts showing the ln IC_50_ of seven NPC cell lines toward four compounds targeting the PI3K/AKT/MTOR pathways, with NPC268 being the most sensitive line relative to other NPC cell lines.

To determine whether any of the genomic/transcriptomic features observed in NPC268 could explain its response to a panel of preselected cancer drugs, we conducted a high-throughput anti-cancer drug screening on late passage NPC268. Six other NPC lines and the NP550 were also included on the screen. We shortlisted 125/339 compounds, where at least one NPC line had an IC_50_ of 1 µmol/L or less. The natural log of the IC_50_ (ln IC_50_) of the 125 compounds for the seven NPC cell lines is depicted in the heat map, whereas the ln IC_50_ of NP550 is plotted in a bar chart next to the heat map for reference (Fig. 7A; Supplementary Dataset S3). The interactive heat map with details on the ln IC_50_ values is accessible via the Morpheus tool (https://software.broadinstitute.org/morpheus/), using JSON file downloadable via figshare: https://doi.org/10.6084/m9.figshare.21747095 (Supplementary Dataset S3). Of the 14 chemotherapy drugs (from 125 compounds), those targeting microtubules, including paclitaxel, docetaxel, vinblastine, and vinorelbine, were potent in all seven NPC lines (with IC_50_ ranging from 0.4 to 200 nmol/L). The other 111 targeted compounds were classified into 16 categories based on the targeted pathways, and the heat map was arranged on the basis of these annotated pathways. Notably, EGFR inhibitors (red color in pathway key), one of the most tested targeted drug classes in clinical trials for NPC, were ineffective in most NPC lines, except for the EBV-negative line HK1. Our results are consistent with the lack of clinical response to these EGFR inhibitors (57). We also found that NPC268 was preferentially sensitive to drugs targeting BCL-2 family proteins (Fig. 7B). NPC268 had the lowest IC_50_ for all three active BCL-2 inhibitors: obatoclax, sabutoclax, and TW37 (Fig. 7C). We demonstrated that high *BCL2L2* gene expression was significantly correlated with the drug response of obatoclax (low ln IC_50_; Pearson *R* = −0.776, *P* = 0.040; Fig. 7D). A similar trend was observed with sabutoclax (Pearson *R* = −0.723, *P* = 0.066) and TW37 (Pearson *R* = −0.643, *P* = 0.119), albeit not statistically significant (Supplementary Fig. S6A and S6B). *BCL2L2,* found on chromosome 14q11.2*,* encodes for the BCL2-like protein 2, which has been reported to be bound by obatoclax. Our results suggest that, for NPC that overexpress *BCL2L2*, drugs targeting these apoptosis regulators may have a potent antitumor effect.

Focusing on drugs targeting the PI3K/AKT/MTOR pathway, we also investigated whether the E17K hotspot mutation in AKT1 of NPC268 (Fig. 7E) affected its response toward drugs targeting these pathways. Figure 7F shows the selected compounds targeting this pathway, and NPC268 is the most sensitive cell line among all NPC lines. Although the AKT1 E17K mutation is rarely reported in NPC (0.6% in a Southern China NPC cohort; ref. 58), this mutation has been used as a biomarker in several clinical trials of AKT inhibitors in solid tumors (AZD5363-Capivasertib, ARQ 092; refs. 52, 59, 60), with an objective response rate of 28.6% reported for metastatic tumors with AKT1 E17K mutation in the NCI-MATCH Subprotocol EAY131-Y trial (52).

## Discussion

The scarcity of cell line models to investigate the molecular mechanisms underlying NPC development underscores the critical need in establishing representative cell line models that are well annotated. In mainland China and Hong Kong, as NPC is dominated (up to 95%) by the undifferentiated subtype, the existing EBV-positive cell lines were all derived from this subtype and little is known about the non-keratinizing differentiated subtype. NPC268 is a unique NPC cell line representing the only available model for the non-keratinizing differentiated subtype (up to 41% in South East Asia countries), where patients have a poorer prognosis compared with the undifferentiated subtype (61). Importantly, NPC268 retained its EBV infection even at late passage with the expression of latent and lytic genes comparable to other EBV-positive lines, and could be induced into the lytic cycle, making it an ideal model to study EBV host genome interaction and the evaluation of novel therapeutics with cytolytic activity. This is exemplified by the retainment of EBV positivity in the late passage NPC268 xenografts, that showed high tumorigenicity while demonstrating presence of permissive infection and lytic reactivation *in vivo*. Interestingly, we also observed a striking increase in proportion of more differentiated and squamous-like cells among the late passage xenografts. As the late passage, NPC268 cells were cultured long term *in vitro* in presence of ROCK inhibitor, we postulated that the withdrawal of ROCK inhibitor *in vivo* that relieved the suppression effect on differentiation and lytic reactivation could have led to the observed phenomena in late passage xenografts.

Considering that NPC268 was derived from a patient of Chinese origin, it is perhaps not surprising to find that NPC268 harbors the oncogenic V-val EBNA1, 30 bp-deleted LMP1 and the more lytic-enhanced Zp-V3 promoter. The V-val EBNA1 has been reported to have increased transcriptional activity, while the 30 bp-deleted LMP1 with 10 amino acids deleted between the carboxyl-terminal activating region 1 (CTAR1) and (CTAR2) domains could increase NFκB reporter activity (62). The Zp-V3 version of the *BZLF1* promoter was found to be overrepresented in NPC (43) and functional studies by Bristol and colleagues confirmed the enhanced lytic reactivation capability of Zp-V3 compared with the prototype Zp-P, raising an interesting postulation that Zp-V3 could contribute to the development of NPC among the Southern Chinese, where the variant is more prevalent (44). In this regard, NPC268 could serve as a useful model to investigate the functional consequences of these high-risk EBV variants in NPC tumorigenesis. Surprisingly, unlike other EBV-positive lines such as NPC43, NPC268 did not harbor the common high-risk variants of *BALF2* that was found in up to 93% of NPC cases in endemic region of China. Whether there is any significant difference of *BALF2* variant frequency among South East Asians NPC and China's NPC, remains to be investigated.

Both karyotype and genomic analyses of NPC268 showed complex chromosomal rearrangements with a high number of SVs, with the most striking being the loss of chromosome 2. Chromosome 2 could be vulnerable to rearrangements, as sequences with high EBV homology were reported on this chromosome in NPC and familial breast cancers (63). Chromothripsis of chromosome 2 and a potentially pathogenic SNV in *PMS2* suggest a high likelihood that the MMR pathway is defective in NPC268. Notably, deficient MMR has been reported in up to 49.3% of NPC cases (64). The identification of specific EBV integration sites could provide insights into the mechanisms by which EBV disrupts host genome stability. Furthermore, NPC268 was found to be the only other NPC cell line thus far (besides NPC43), which represents the recently described HypoNPC subtype (found in about 20% of patients with NPC), which showed an increased APOBEC-associated mutational signature (34). NPC268 has high genome instability (MMR defective, high TMB) and resembles HypoNPC with a low level of the *CD74* immune exhaustion marker, as well as upregulation of genes related to the immune pathway. It also has the highest level of *CXCL10* expression, the chemokine that overexpressed in MMR defective cancers, which were shown to be essential for recruitment and activation of CD8^+^ T cells (65). Furthermore, NPC268 tumors showed strong LCA/CD45 staining, revealing a high level of tumor-infiltrating lymphocytes (Fig. 1D). Taken together, we postulate that NPC268 may be amenable to immunotherapy. Work to use NPC268 as a model to evaluate cancer vaccines is currently ongoing.

High-throughput drug screening could serve as the powerhouse to fuel new drug discovery or drug repurposing; however, such data have not been available for NPC. Our study offers the largest drug response data to date for NPC cell lines, and combining this with deep genomic characterization of these cell lines by us and others, we identified classes of drugs with potential biomarkers, warranting further investigation in more NPC preclinical models. The heterogeneity in the epigenetic landscape of NPC and distinct drug response patterns underscores the importance of continued efforts in establishing representative NPC models.

Taken together, NPC268 could serve as a valuable model for the *in vitro* and *in vivo* study of NPC and EBV, empowering our improved understanding of the role of EBV in NPC pathogenesis and enabling preclinical assessment of novel therapeutic strategies for the treatment of NPC.

## Supplementary Material

Supplementary Methods Supplementary Methods

Supplementary Table 1 Primer list and sequences

Supplementary Table 2 List of 211 published EBV genomes used in this study

Supplementary Table 3 Short Tandem Repeats (STR) profiling of NPC268 cell lines at early and late passages confirmed authenticity with patient's buffy coat DNA

Supplementary Table 4 Antibody and probe list for immunohistochemistry

Supplementary Table 5 Antibodies used for western blotting

Supplementary Table 6 Comparison of variants identified between NPC268 and C666-1

Supplementary Table 7 List of SNVs found in mutational cancer driver genes in NPC268

Supplementary Table 8 Genes affected by substantial copy number variation in NPC268

Supplementary Table 9 Summary table comparing NPC268 with other three EBV-positive lines

Supplementary Figure 1 Karyotype analysis of NPC268 and confirmation of epithelial origin.

Supplementary Figure 2 EBV in NPC268 can be induced into lytic phase using various chemical inducers.

Supplementary Figure 3 NPC268 is capable of anchorage-independent growth and is tumorigenic in vivo.

Supplementary Figure 4 The genomically unstable NPC268 showed high levels of gamma H2A.X and LINE1 RNA

Supplementary Figure 5 Activation of immune-related pathway such as the interferon response pathway.

Supplementary Figure 6 Correlation analyses of BCL2L2 gene expression with its inhibitors.

Supplementary Figure 7 Uncropped western blot images
