# Supplementary material for: Establishment and Characterization of an Epstein-Barr Virus–positive Cell Line from a Non-keratinizing Differentiated Primary Nasopharyngeal Carcinoma
Source: Cancer Res Commun. 2024 Mar 4;4(3):645–59. doi: 10.1158/2767-9764.CRC-23-0341 (PMC10911800; doi:10.1158/2767-9764.CRC-23-0341)
Supplement: Supplementary Methods [file crc-23-0341-s01.docx]

# SUPPLEMENTARY METHODS

## NPC tissue collection, cell line establishment and maintenance

The resected NPC tissue used for the NPC268 cell line establishment was collected from a consented patient with institutional review board approval. The tissue was collected in DMEM/F12 supplemented with 2% IU penicillin/streptomycin and 2% fungizone, and additional tissue samples were snap-frozen in liquid nitrogen for cryo-preservation and used for WGS, as described below.

The collected tissue was first washed with absolute ethanol for 10 seconds, and then twice with phosphate-buffered saline (PBS) under sterile conditions. The tissue was minced into smaller pieces and transferred into trypsin explant solution, containing 2.5mL trypsin and 7.5mL PBS, and incubated at 4°C overnight. The next day, the tissue in the trypsin explant solution was incubated at 37°C for 30 min and further dissociated by vigorous pipetting. Cells were spun down at 2,000 rpm for 15 min and explanted onto a culture dish seeded with a layer of irradiated 3T3-J2 feeder cells. Tissue explants were cultured in FAD medium [DMEM/F12 (1:1) with insulin (5μg/mL), adenine (0.18 mM), hydrocortisone (0.4μg/mL), and triiodothyronine (2nM)] supplemented with 10µM Y-27632, 2% penicillin/streptomycin (Pen/Strep), and 2% fungizone. The cells were monitored daily, and the feeders were changed weekly to facilitate epithelial cell outgrowth from the explant. After the initial passages, which took up to six months to reach confluency, the established NPC268 cell line was grown in the absence of feeders, and the Y-27632 concentration was reduced from 10µM to 4µM. From passage 10 onwards, the cell line was continuously propagated to monitor its growth dynamics and population doubling time. gDNA and RNA samples were collected every five passages for EBV copy number monitoring and to determine the expression of EBV-related genes.

## Cell line authentication by short tandem repeat genotyping

NPC268 was authenticated to DNA obtained from the buffy coat of the patient. In brief, polymerase chain reaction (PCR) was used to amplify at least eight short tandem repeat (STR) loci, including the gender determination marker amelogenin, either in the PowerPlex 16HS Assay (Promega, USA) or GenePrint® 24 System (Promega, USA). The PCR product was further processed using an ABI PRISM® 3100 Genetic Analyzer and analyzed using either GeneMarker (SoftGenetics, USA) or GeneMapper® v5.0 software (Applied Biosystems, Foster City, CA, USA). DNA profiles of the cell line, buffy coat, and tumor DNA were compared according to the recommendations of the International Cell Line Authentication Committee (ICLAC) guidelines.

## Cell block preparation

Cell suspensions (5–10 million cells) were centrifuged and washed with PBS. The cells were then fixed in 10% neutral buffered formalin for 24 hours at room temperature on a rotator. After fixation, cells were washed with PBS and pelleted. The cell pellet was resuspended in 200μl of pre-warmed agar solution (0.8% agarose in PBS), mixed evenly, and then transferred onto a plastic mold or parafilm to solidify at 4°C. The agar blocks were further processed according to the histological sample preparation protocol.

## Histological sample preparation and immunohistochemical staining

The protocols were adapted from Hoe et al. (2017) [29]. Xenograft tissues were formalin-fixed for 24 hours, processed using an automated tissue processor Leica ASP300 S (Leica Biosystems, Melbourne, Australia), paraffin-embedded, and sectioned at 3μm thickness. The sections were subjected to hematoxylin and eosin (H&E), immunohistochemical (IHC), and *in situ* hybridization (ISH) staining. H&E staining was performed using an autostainer (Leica XL; Leica Biosystems). All pre-staining steps (baking, dewaxing, rehydrating, and antigen retrieval) and subsequent IHC or ISH staining were performed on the BOND-MAX Fully Automated IHC and ISH Staining System (Leica Biosystems) using BOND automated detection systems. Sections were stained using modified protocols for BOND Polymer Refine Detection or BOND Intense R Detection Systems (Leica Biosystems, UK). Marker expression was visualized using diaminobenzidine (DAB) chromogen, and hematoxylin was used as a counterstain. All staining steps were followed by washing with washing buffer or deionized water. After staining, the samples were dehydrated by passing in absolute alcohol, followed by clearing in xylene. Finally, the slide samples were mounted on coverslips using mounting medium. The antibodies and probes used, their respective detection systems, and antigen retrieval are listed in Supplementary Table 4.

## EBV lytic induction and B cell infection using EBV from NPC268

To induce the EBV lytic cycle, NPC268 cells grown to ~50% confluence were transfected with the BZLF1 expressing plasmid p509 (a kind gift from Prof. Wolfgang Hammerschmidt, German Research Center for Environmental Health, Munich, Germany) using the Jetprime transfection reagent (Polyplus-transfection, Illkirch, France), and fresh media was replaced after 24 hours of transfection. In parallel, lytic reactivation of EBV was also carried out with suberoylanilide hydroxamic acid (SAHA) (10µM), sodium butyrate (NaBu) (6mM) or tiglian 12-O-tetradecanoylphorbol-13-acetate (TPA) (40ng/ml) for 24, 48, or 72 hours. DMSO was used as the negative control. Following transfection or treatment, cells were harvested to collect protein lysates to determine the expression of lytic markers Zta and Ea-D.

After 96 hours post-transfection with BZLF1-expressing plasmid p509, the conditioned medium of NPC268 was collected, centrifuged, and filtered to remove cell debris. The supernatant was then subjected to ultracentrifugation at 20,000 rpm for 4 hours at 4°C to pellet down the EBV viral particles. The pellet was resuspended in fresh RPMI-1640 medium at 1/20 of its original volume to concentrate the EBV virus. About 900ul of EBV concentrate was used to infect EBV-negative Akata cells (kindly provided by Professor Kenzo Takada, Hokkaido University, Japan), as previously described [23]. After 48 hours, Akata cells were harvested, and complementary DNA was used for qPCR to determine EBV gene expression.

## Lysates preparation and western blotting

Total cell lysates (TCL) were extracted using RIPA buffer (50 mM Tris pH 8, 1% (v/v) NP-40, 0.5% (w/v) sodium deoxycholate, 0.1% (w/v) SDS, and 150mM NaCl supplemented with Halt Protease and Phosphatase Inhibitor Cocktail (Pierce Biotechnology, Rockford, IL, USA) on ice. TCL were collected and quantified using BCA method (Thermo Fisher Scientific). About 20μg of TCL was resolved by SDS-PAGE and transferred onto PVDF membranes (Millipore, Burlington, MA, USA) at 400mA for 1 hour on ice. The membranes were blocked with 5% (w/v) milk in TBST (0.1% [v/v] Tween 20) for 1 h. After brief washing in TBS supplemented with 0.1% Tween 20 (Sigma Aldrich; TBST), the membranes were probed with primary antibodies at a 1:500 dilution in 1% (w/v) bovine serum albumin overnight at 4°C. The membranes were washed thrice in TBST for 10 min each. The membranes were then incubated with the corresponding secondary antibody (1:10,000 dilution in 5% milk) (Southern Biotech, USA) for 1 hour at room temperature. This was followed by washing three times in TBST for 10 min each, signal detection using the Immobilon Forte Western HRP substrate (Merck Millipore, Germany), and visualization using the Azure c300 imaging system (Azure Biosystems, USA). To normalize for loading, the blots were re-probed with a β-actin monoclonal antibody (1:5000 dilution in 1% bovine serum albumin) and processed as described above. The list of antibodies used is provided in Supplementary Table 5.

## Total RNA extraction and quantitative reverse transcription PCR (qRT-PCR)

Total RNA was extracted using TRIzol reagent (Thermo Fisher Scientific, Waltham, MA, USA), according to the manufacturer’s instructions. 1µg RNA was reverse transcribed into complementary DNA (cDNA) using a High-Capacity cDNA Reverse Transcription Kit (Applied Biosystems, Waltham, MA, USA). Real-time quantitative PCR was conducted using 1µL of 10x diluted cDNA with PowerUp SYBR Green Master Mix (Applied Biosystems) and the relevant primers in a 7500 Real-Time PCR System (Applied Biosystems). All qPCR reactions were run in triplicates, twice. Ribosomal protein L13 (RPL13) was used as an endogenous reference control for normalization and the relative expression level of each EBV gene was determined using the 2^-ΔΔCt^ method. The sequences of all the primers used are shown in Supplementary Table 1.

For LINE RNA detection and amplification, to remove single-stranded RNA and enrich for double-stranded RNA (dsRNA), 1µg total RNA was treated with RNase If (NEB-M0243) for 37°C – 15 minutes, followed by heat inactivation at 70°C for 20 minutes. The dsRNA-enriched RNA was then converted to cDNA and used for qPCR as described above. Normalization was done using RPL13 Ct derived from corresponding non-RNAse treated sample.

## Whole-genome bisulfite sequencing (WGBS)

The gDNA of cell lines was processed for bisulfite conversion and library preparation, and WGBS was performed and analyzed as described in [34]. Trimmomatic (v.033) was used to remove adapter sequences, poor-quality bases, and reads shorter than 30 base pairs in the raw FASTQ files [62]. The trimmed reads were aligned to the reference genome composed of the human genome (hg19) and EBV genome (NC_007605). Duplicate reads were detected and removed using Picard (v2.17.4). The methylation ratio was quantified at base-pair resolution using BSMAP (v2.9) [63]. The methylation ratio of a CpG site/region is the number of cytosine reads divided by the sum of the cytosine and thymine reads. FastQC (v0.11.8), Picard, and in-house scripts were used to examine the quality of the data, including the unique mapping rate, bisulfite conversion efficiency, and coverage, as previously described [34]. The human genome was segmented using a Methyl Kit (v.1.16.1) for global methylation pattern visualization [35].

## Soft agar assay

The anchorage-independent growth ability of the NPC268 cells was assessed using the soft agar assay. The assay was set up in a 6-well culture plate using a 0.3% agarose base and a 0.15% agarose layered on top with 5 × 10^4^ cells. After 28 days of incubation, colonies with a diameter greater than 100 μm from five representative fields were included and analyzed using the ImageJ software (National Health Institute (NIH), USA).

## Immunofluorescence staining

NPC268 cells were seeded onto round sterilized coverslip in 12-well plate. Next day, cells were fixed with 4% paraformaldehyde for 15 minutes at room temperature. Fixative was then rinsed and cells were washed with PBS. Blocking was performed for an hour with 1% bovine serum albumin, then probed with primary antibody against phospho-Histone H2A.X (20E3, #9718, Cell Signaling Technologies) at 1:250, overnight at 4°C. Next day, cells were washed and probed with 1:500 anti-rabbit Alexa Fluor 555 secondary antibody (A32732, Invitrogen) for an hour. Cells were then stained with Hoechst dye for 35 minutes, washed and mounted with Vectashield mounting medium H-1000. Images were taken with Axio Imager M2 microscope (Zeiss) at 40x magnification.

**References:**

1. Hoe, S.L.L., et al., *CD24, CD44 and EpCAM enrich for tumour-initiating cells in a newly established patient-derived xenograft of nasopharyngeal carcinoma.* Scientific Reports, 2017. **7**(1): p. 12372.

2. Lin, W., et al., *Establishment and characterization of new tumor xenografts and cancer cell lines from EBV-positive nasopharyngeal carcinoma.* Nat Commun, 2018. **9**(1): p. 4663.

3. Ka-Yue Chow, L., et al., *Epigenomic landscape study reveals molecular subtypes and EBV-associated regulatory epigenome reprogramming in nasopharyngeal carcinoma.* EBioMedicine, 2022. **86**: p. 104357.

4. Bolger, A.M., M. Lohse, and B. Usadel, *Trimmomatic: a flexible trimmer for Illumina sequence data.* Bioinformatics, 2014. **30**(15): p. 2114-20.

5. Xi, Y. and W. Li, *BSMAP: whole genome bisulfite sequence MAPping program.* BMC Bioinformatics, 2009. **10**: p. 232.

6. Akalin, A., et al., *methylKit: a comprehensive R package for the analysis of genome-wide DNA methylation profiles.* Genome Biol, 2012. **13**(10): p. R87.
