# Supplementary material for: Establishment and Characterization of an Epstein-Barr Virus–positive Cell Line from a Non-keratinizing Differentiated Primary Nasopharyngeal Carcinoma
Source: Cancer Res Commun. 2024 Mar 4;4(3):645–59. doi: 10.1158/2767-9764.CRC-23-0341 (PMC10911800; doi:10.1158/2767-9764.CRC-23-0341)
Supplement: Supplementary Figure 1 — Karyotype analysis of NPC268 and confirmation of epithelial origin. [file crc-23-0341-s11.pdf]

Supplementary Figure 1

(A) **EARLY – Clone 1**

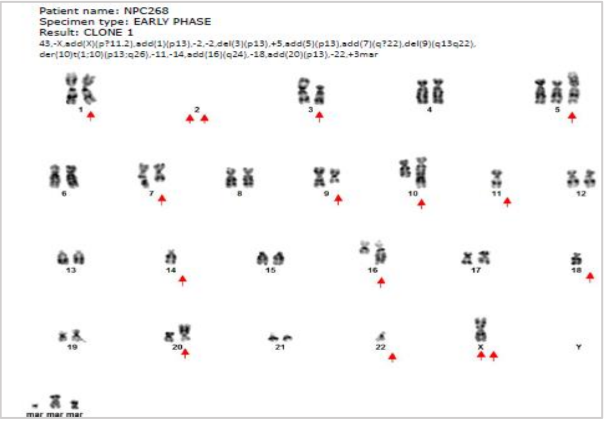

(B) **EARLY – Clone 2**

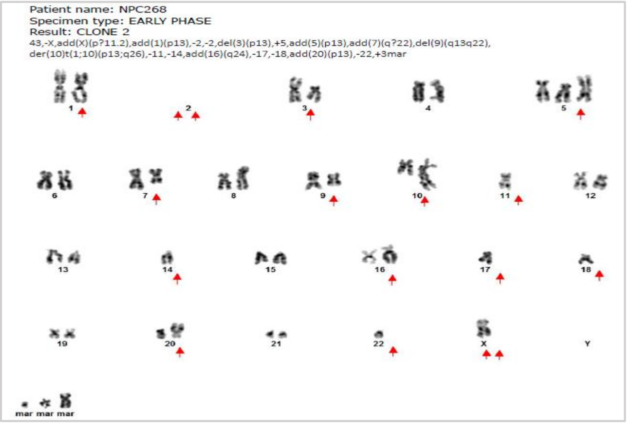

(C) **EARLY – Clone 3**

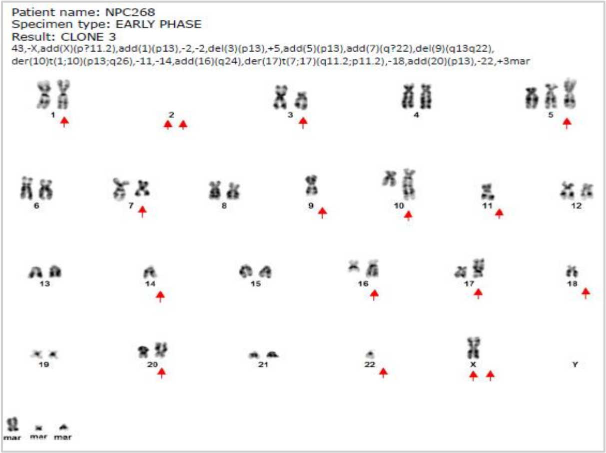

(D) **EARLY – Clone 4**

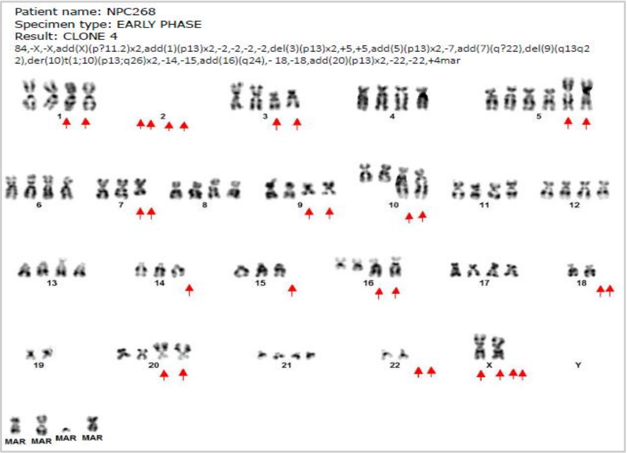

(E) **LATE – Clone 1**

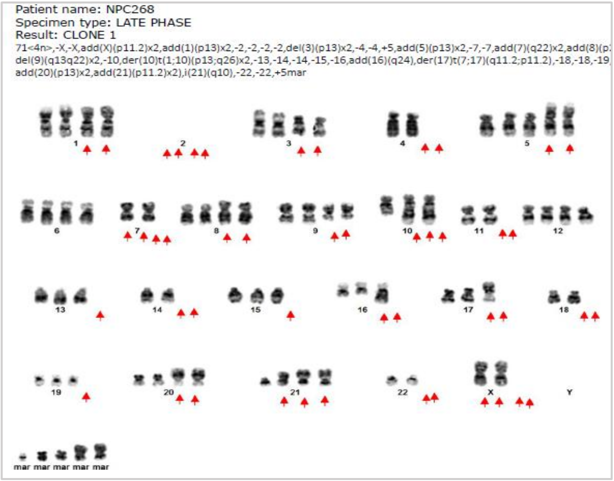

(F) **LATE – Clone 2**

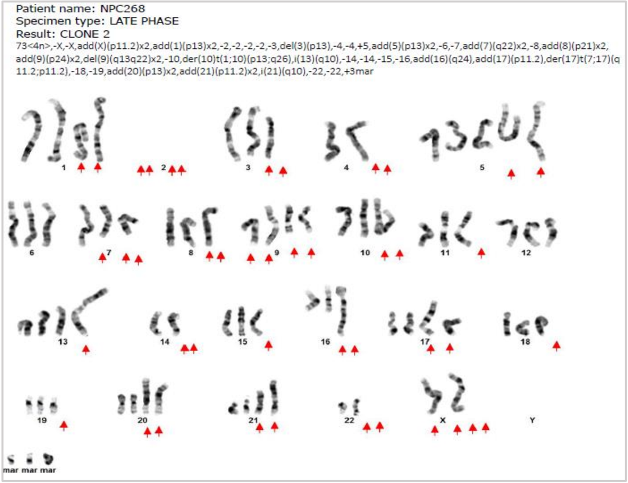

(G) LATE – Clone 3

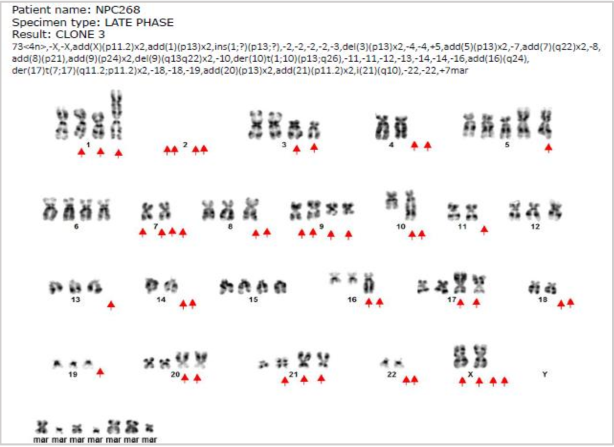

(H)

H&E Pan-cytokeratin

NPC268 p24

NPC268 p108

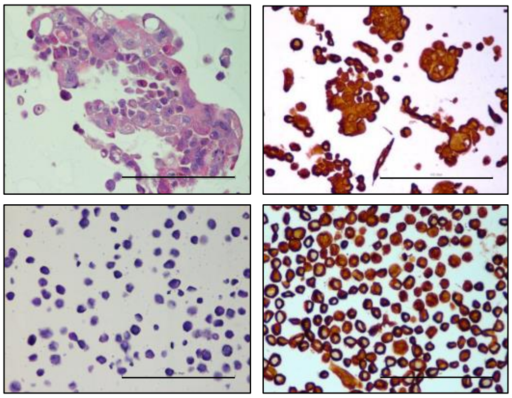

Supplementary Figure 1. Karyotype analysis of NPC268 and confirmation of epithelial origin.

(A-D) Metaphases of four major clones at early passage (p24) showed hypodiploid or hypotetraploid. **(E-G)** At late passage (p108), metaphases of all three major clones were of hypotetraploids. Structural abnormalities in chromosomes Xp, 1p, 3p, 5p, 7q, 8p, 9q, 16q, 17p, 20p and 21p, unbalanced 1;10 and 7;17 translocations, an isochromosome 21q and the addition of several marker chromosomes were observed. Four of these metaphases also had an isochromosome 13q while another five metaphases had an insertion of unknown material onto the short arm of one chromosome 1. **(H)** Epithelial origin of NPC268 cell line was confirmed using immunohistochemistry (IHC) staining of pan-cytokeratin marker. Scale bar: 100µm.
