# Supplementary material for: Establishment and Characterization of an Epstein-Barr Virus–positive Cell Line from a Non-keratinizing Differentiated Primary Nasopharyngeal Carcinoma
Source: Cancer Res Commun. 2024 Mar 4;4(3):645–59. doi: 10.1158/2767-9764.CRC-23-0341 (PMC10911800; doi:10.1158/2767-9764.CRC-23-0341)
Supplement: Supplementary Figure 2 — EBV in NPC268 can be induced into lytic phase using various chemical inducers. [file crc-23-0341-s12.pdf]

Supplementary Figure 2

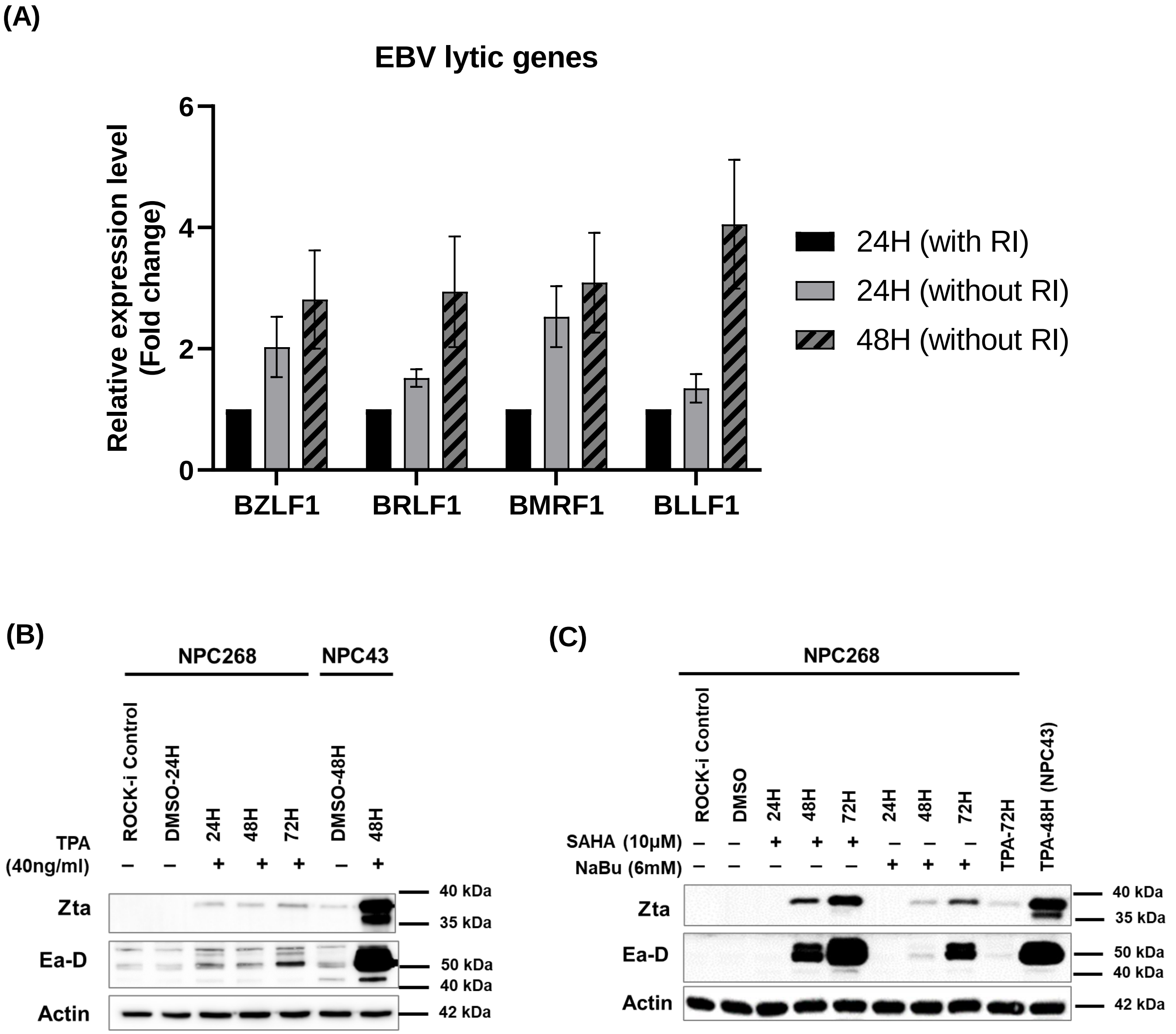

Supplementary Figure 2. EBV in NPC268 can be induced into lytic phase using various chemical inducers.

(A) Rock inhibitor (Y-27632) helps to suppress EBV lytic reactivation during cell line establishment. Upon removal of Y-27632 from early passage NPC268, we observed gradual re-expression of *BZLF1*, *BRLF1*, *BMRF1* and *BLLF1.* Data shown is mean $\pm$S.E.M, (n = 2 independent experiments with technical triplicates). **(B)** TPA can be used to induce expression of lytic proteins Zta and Ea-D in NPC268, TPA-treated NPC43 was used as positive control. **(C)** SAHA and NaBu were also tested for their abilities to induce lytic reactivation in NPC268, SAHA showed the most potent effect, comparable with the effect of TPA on NPC43.
