# Supplementary material for: Establishment and Characterization of an Epstein-Barr Virus–positive Cell Line from a Non-keratinizing Differentiated Primary Nasopharyngeal Carcinoma
Source: Cancer Res Commun. 2024 Mar 4;4(3):645–59. doi: 10.1158/2767-9764.CRC-23-0341 (PMC10911800; doi:10.1158/2767-9764.CRC-23-0341)
Supplement: Supplementary Figure 3 — NPC268 is capable of anchorage-independent growth and is tumorigenic in vivo. [file crc-23-0341-s13.pdf]

Supplementary Figure 3

(A)

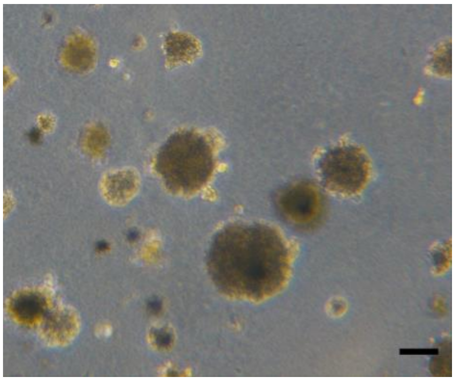

(B)

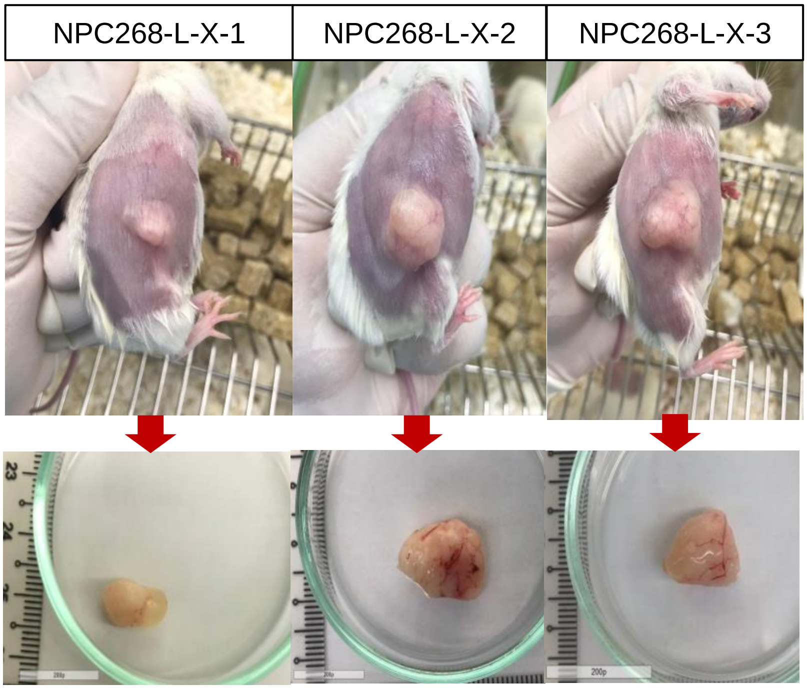

Supplementary Figure 3. NPC268 is capable of anchorage-independent growth and is tumorigenic *in vivo*.

(A) NPC268 cell line is capable of anchorage-independent growth, as demonstrated by the soft agar assay. Spheroids of greater than 100µm diameter were seen after four weeks. **(B)** NPC268 can form tumors in NOD/SCID gamma (NSG) mice. Two out of three xenografts (L-X-2, L-X-3) form tumor of 1000mm^3 in about 43 and 46 days respectively.
