# Supplementary material for: Establishment and Characterization of an Epstein-Barr Virus–positive Cell Line from a Non-keratinizing Differentiated Primary Nasopharyngeal Carcinoma
Source: Cancer Res Commun. 2024 Mar 4;4(3):645–59. doi: 10.1158/2767-9764.CRC-23-0341 (PMC10911800; doi:10.1158/2767-9764.CRC-23-0341)
Supplement: Supplementary Figure 4 — The genomically unstable NPC268 showed high levels of gamma H2A.X and LINE1 RNA [file crc-23-0341-s14.pdf]

Supplementary Figure 4

(A)

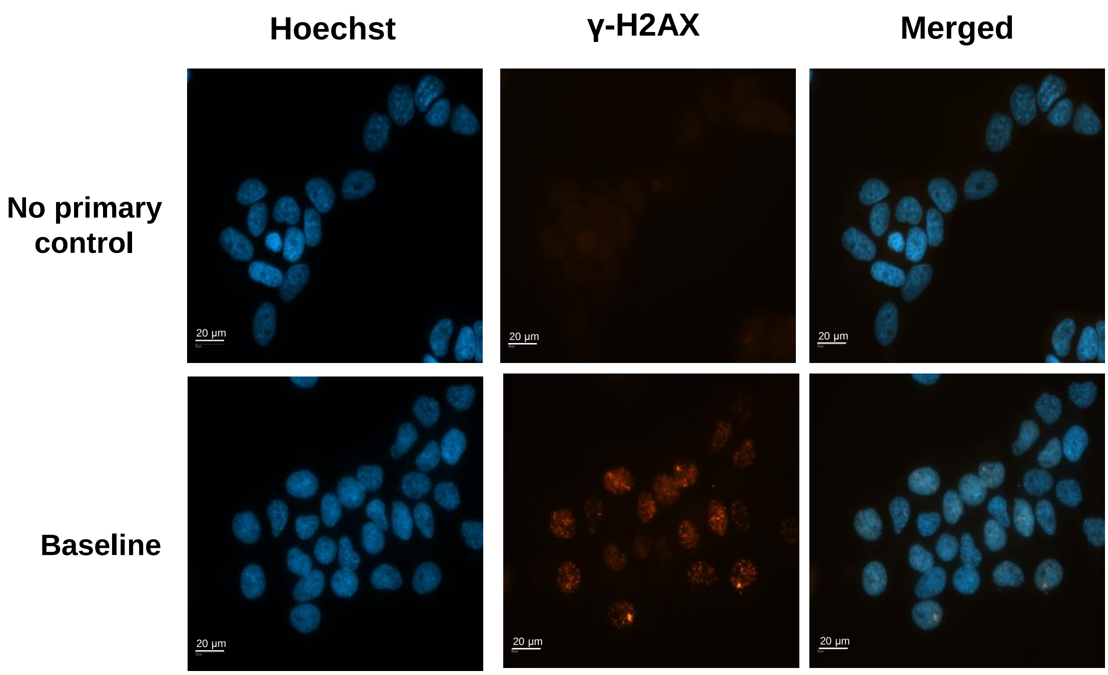

(B)

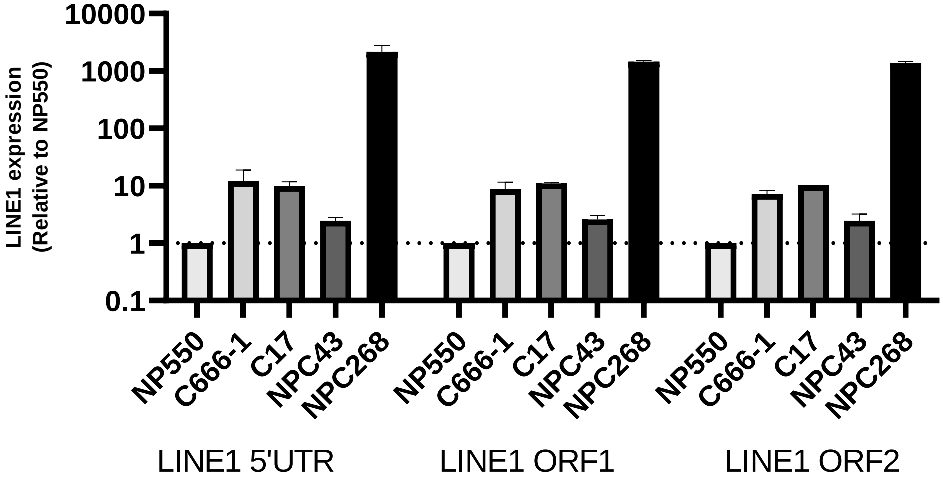

Supplementary Figure 4. The genomically unstable NPC268 showed high levels of gamma H2A.X and LINE1 RNA. (A) gamma-H2AX can be readily detected with immunofluorescence in NPC268 cells at baseline. **(B)** qPCR amplification of cDNA derived from RNAse I-treated RNA showed that NPC268 overexpresses LINE1. Data are shown as mean ± SD (n = 2 independent experiments with technical triplicates).
