# Supplementary material for: Establishment and Characterization of an Epstein-Barr Virus–positive Cell Line from a Non-keratinizing Differentiated Primary Nasopharyngeal Carcinoma
Source: Cancer Res Commun. 2024 Mar 4;4(3):645–59. doi: 10.1158/2767-9764.CRC-23-0341 (PMC10911800; doi:10.1158/2767-9764.CRC-23-0341)
Supplement: Supplementary Figure 5 — Activation of immune-related pathway such as the interferon response pathway. [file crc-23-0341-s15.pdf]

Supplementary Figure 5

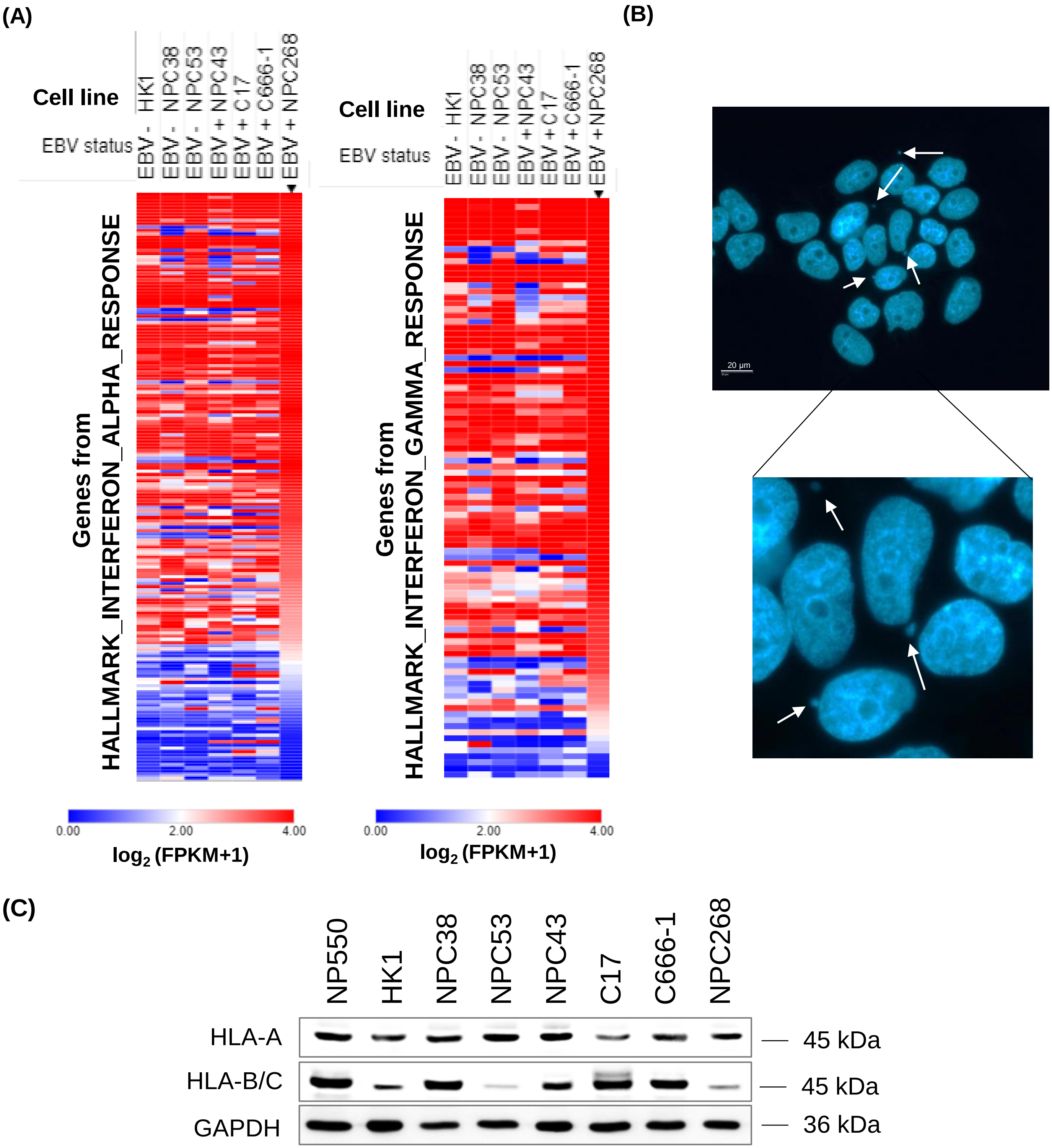

Supplementary Figure 5. Activation of immune-related pathway such as the interferon response pathway. **(A)** Heatmap of gene expression of members of the HALLMARK_INTERFERON_ALPHA_ RESPONSE and HALLMARK_INTERFERON_GAMMA _RESPONSE gene sets for the NPC cell lines. **(B)** Presence of cytoplasmic DNA can be detected as micronuclei in the immunofluorescence-based nucleus staining with Hoechst dye. **(C)** Western blot showing the expression of HLA-A and HLA-B/C in NPC268 and other NPC cell lines.
