# Supplementary material for: Establishment and Characterization of an Epstein-Barr Virus–positive Cell Line from a Non-keratinizing Differentiated Primary Nasopharyngeal Carcinoma
Source: Cancer Res Commun. 2024 Mar 4;4(3):645–59. doi: 10.1158/2767-9764.CRC-23-0341 (PMC10911800; doi:10.1158/2767-9764.CRC-23-0341)
Supplement: Supplementary Figure 6 — Correlation analyses of BCL2L2 gene expression with its inhibitors. [file crc-23-0341-s16.pdf]

Supplementary Figure 6

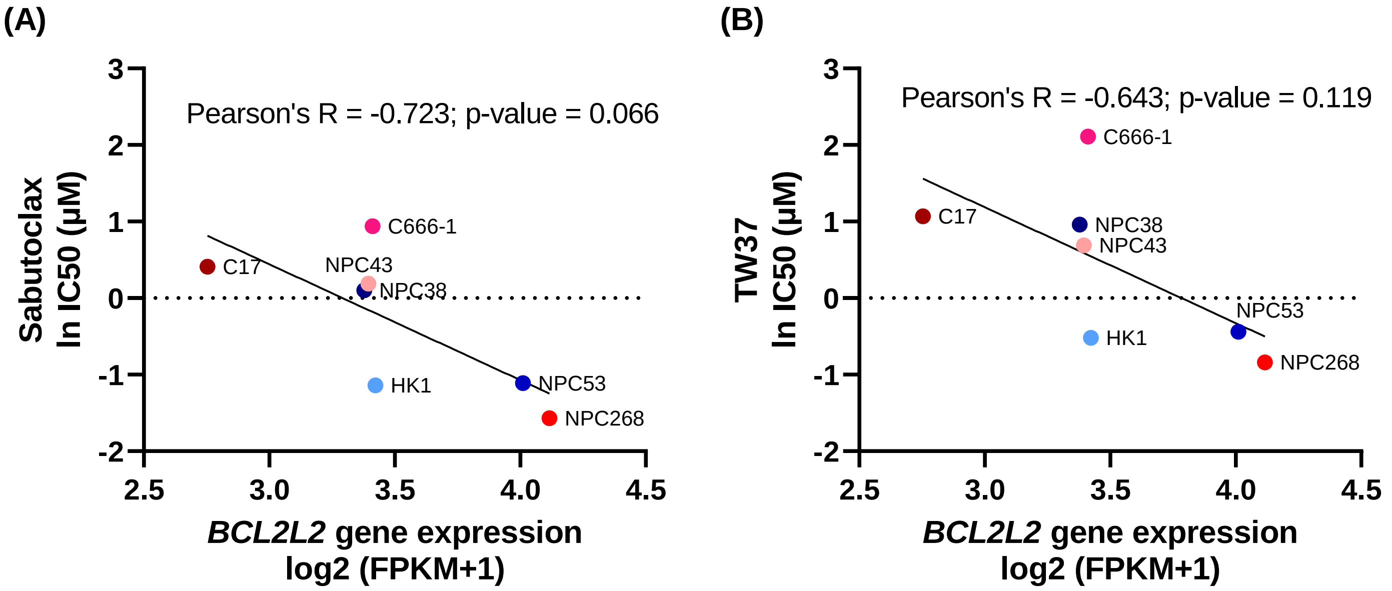

Supplementary Figure 6. Correlation analyses of *BCL2L2* gene expression with its inhibitors. *BCL2L2* expression correlated with sensitivity towards **(A)** sabutoclax (Pearson's R = -0.723, p =0.066) and **(B)** TW37 (Pearson's R = -0.643, p =0.119), albeit not statistically significant.
