# Supplementary material for: Establishment and Characterization of an Epstein-Barr Virus–positive Cell Line from a Non-keratinizing Differentiated Primary Nasopharyngeal Carcinoma
Source: Cancer Res Commun. 2024 Mar 4;4(3):645–59. doi: 10.1158/2767-9764.CRC-23-0341 (PMC10911800; doi:10.1158/2767-9764.CRC-23-0341)

Supplementary Figure 7 – Uncropped western blot images

Figure 2E

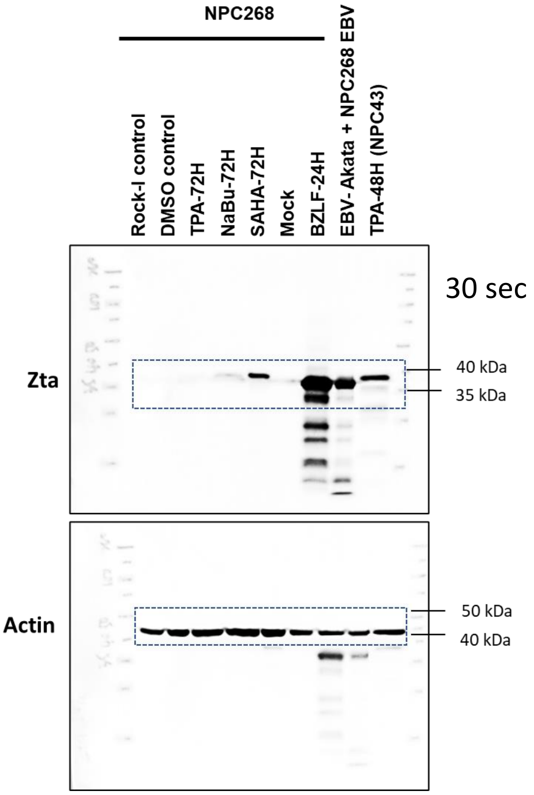

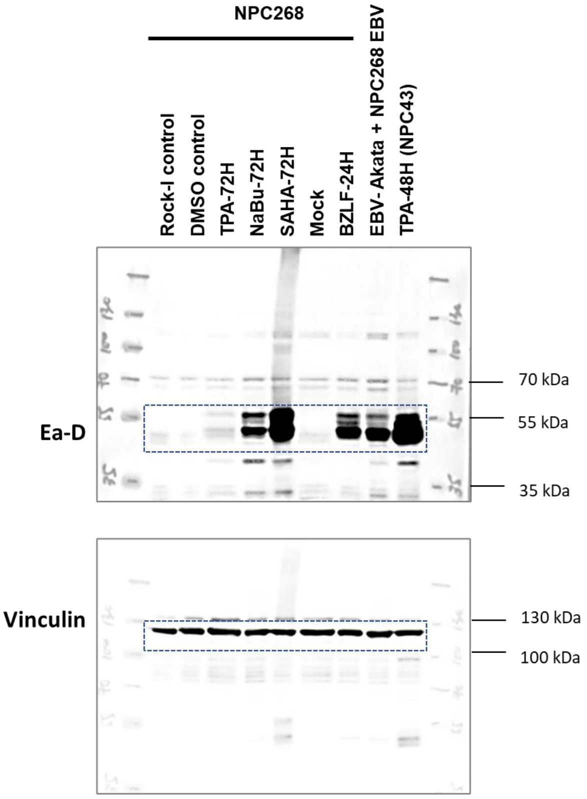

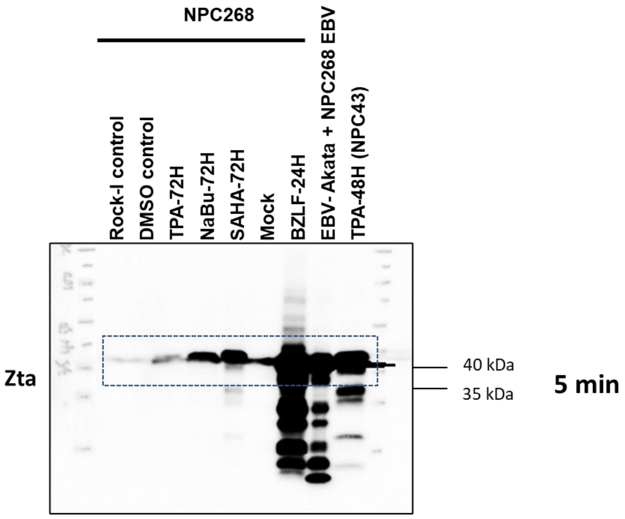

Supplementary Figure 7 – Uncropped western blot images

Supp. Figure 2A

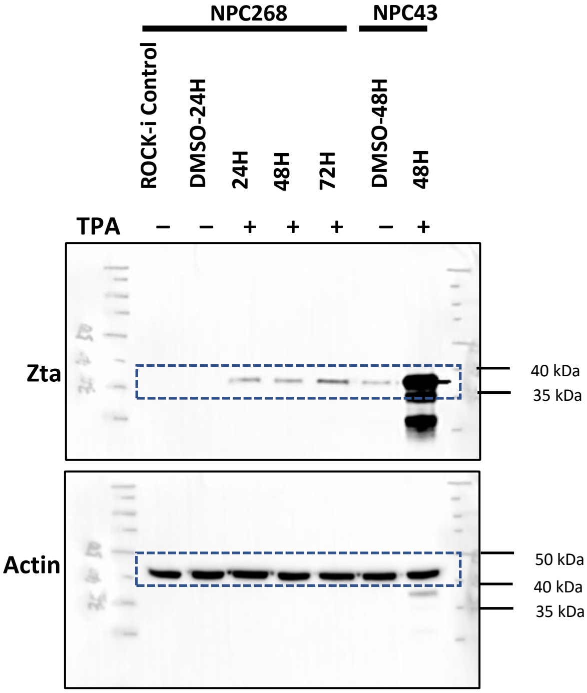

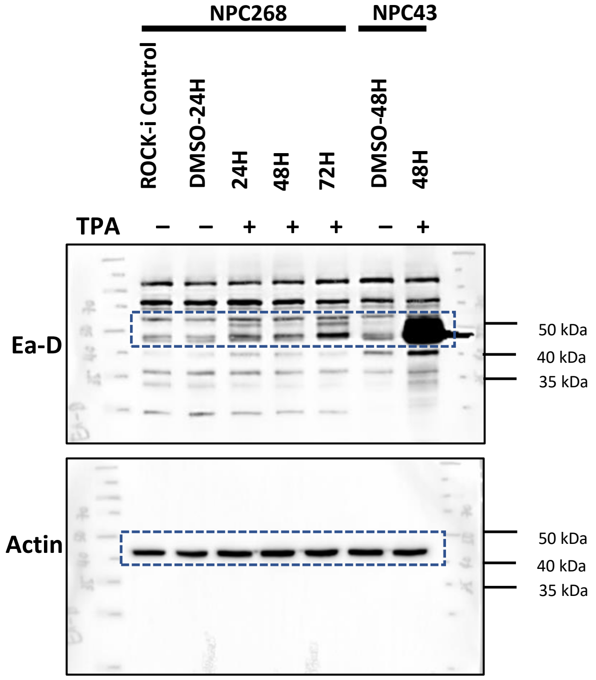

Supp. Figure 2B

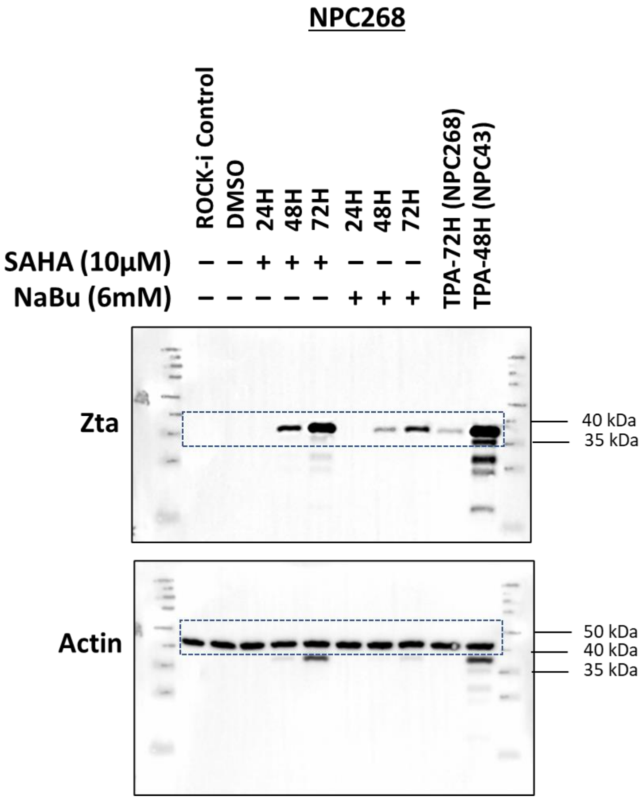

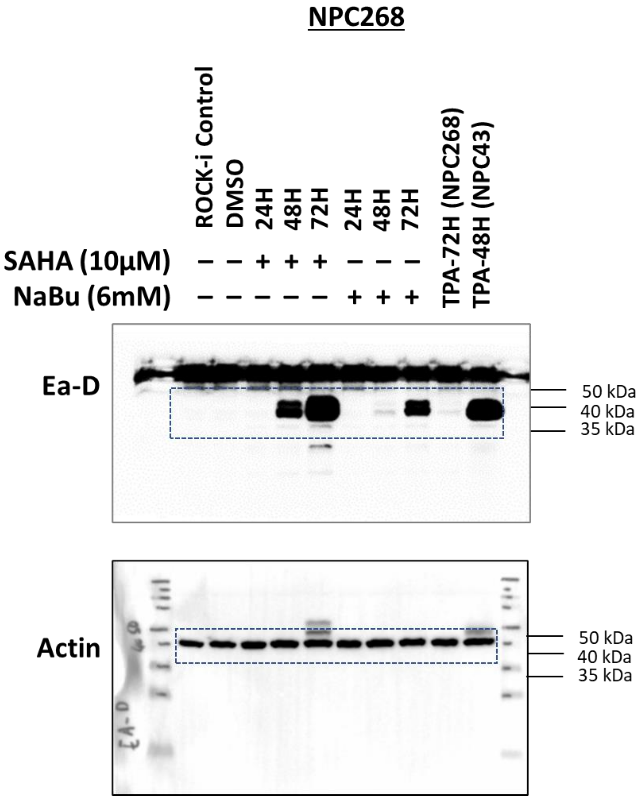

Supplementary Figure 7 – Uncropped western blot images

Supp. Figure 5C

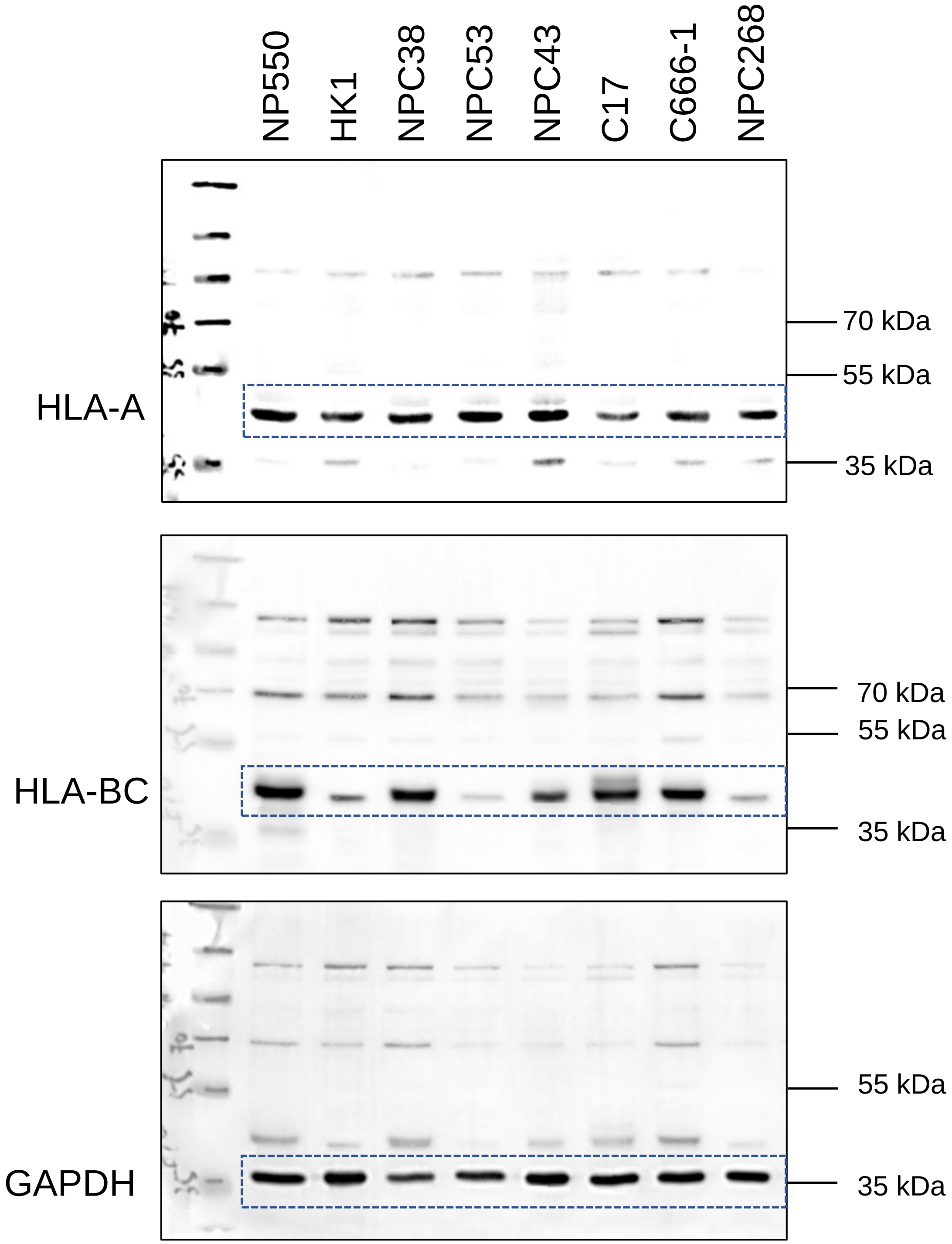

Supplement: Supplementary Figure 7 — Uncropped western blot images [file crc-23-0341-s17.pdf]
